# Effects of Early Intervention with Sodium Butyrate on Gut Microbiota and the Expression of Inflammatory Cytokines in Neonatal Piglets

**DOI:** 10.1371/journal.pone.0162461

**Published:** 2016-09-09

**Authors:** Jumei Xu, Xue Chen, Shuiqing Yu, Yong Su, Weiyun Zhu

**Affiliations:** Jiangsu Key Laboratory of Gastrointestinal Nutrition and Animal Health, College of Animal Science and Technology, Nanjing Agricultural University, Nanjing, 210095, China; National Institute for Agronomic Research, FRANCE

## Abstract

Butyrate in the gut of animals has potential properties including regulating the innate immune, modulating the lipid metabolism, and protecting gut healthy. So far, only limited information on the impact of butyrate on the neonatal is available. This study aimed to investigate effects of oral administration of sodium butyrate (SB) on gut microbiota and the expression of inflammatory cytokine in neonatal piglets. Ten litters of crossbred newborn piglets were randomly allocated to the SB and control (CO) groups, each group consisted of five litters (replicates). Piglets in the SB group were orally administrated with 7 to 13 ml sodium butyrate solution (150 mmol/l) per day from the age of 1 to 7 days, respectively; piglets in the CO group were treated with the same dose of physiological saline. On days 8 and 21 (of age), gut digesta and tissues were collected for the analysis of microbiota, butyrate concentration and gene expression of inflammatory cytokine. Results showed that there was no difference in the butyrate concentration in the gut of piglets on days 8 and 21 between two groups. Real-time PCR assay showed that SB had no effect on the numbers of total bacteria in the stomach, ileum, and colon. MiSeq sequencing of the V3-V4 region of the *16S rRNA* gene revealed that SB increased the richness in the stomach and colon, and the diversity of colonic microbiota on day 8 (*P* < 0.05). Genera *Acinetobacter*, *Actinobacillus*, *Facklamia*, *Globicatella*, *Kocuria*, *Rothia*, unclassified Leptotrichiaceae, unclassified Neisseriaceae, and unclassified Prevotellaceae in the stomach were increased in relative abundance by SB treatment, whereas the abundances of *Lactobacillus* decreased on day 8 (*P* < 0.05). At the genus and operational taxonomic unit (OTU) levels, SB had low impact on bacterial community in the ileum and colon on days 8 and 21. SB treatment decreased the expression of *IL-6*, *IL-8*, *IFN-γ*, *IL-10*, *TGF-β*, and *histone deacetylase 1* (*HDAC1*) in the ileum of piglets on day 8 (*P* < 0.05). SB treatment down-regulated the expression of *IL-8*, *IFN-γ*, and *IL-1β* on day 21 (*P* < 0.05). Correlation analysis on the combined datasets revealed some potential relationships between gut microbiota and the expression of inflammatory cytokines. The results show that early intervention with sodium butyrate can modulate the ileum inflammatory cytokine in neonatal piglets with low impact on intestinal microbial structure, which suggests oral administration of SB may have a benefit role in the health of neonatal piglets.

## Introduction

The survival rate of piglets directly affects the development of pig industry. Newborn piglets mainly rely on maternal antibody to against a great number of entero- and bronchopulmonary pathogenic organisms at first week after birth [1, 2]. However, with fewer antibodies in the breast milk, the intestinal mucosal immune seems to play a role in maintaining intestinal health of piglets. Increasing studies based on the germ-free gut have provided clear evidence that the gut microbiota is instrumental in promoting the development of both the gut and systemic immune systems [3]. Early microbial exposure of the gut is thought to dramatically reduce the incidence of inflammatory, autoimmune, and atopic diseases [4–7]. The early colonization of gut microbiota plays a fundamentally important role in the development of intestinal function and the innate immune system [5, 8, 9].

Butyrate, a short-chain fatty acid, is an end-product of intestinal microbial fermentation of mainly dietary fiber. Butyrate is an important energy source for intestinal epithelial cells and can increase the proliferation index in the intestinal crypts [10]. Particularly, butyrate has potential properties of anticarcinogenic and anti-inflammatory [11], affecting the intestinal barrier and playing a role in satiety and oxidative stress. Butyrate was widely used on animal production for its anti-bacteria and anti-inflammatory properties. Galfi and Bokori showed that the inclusion of sodium butyrate (SB) in the diet significantly increased the body weight gain, feed utilization and composition of intestinal microbiota in growing pigs [12]. However, a previous study showed that intestinal counts of clostridia, enterobacteriaceae, and lactic acid bacteria as well as intestinal mucosal morphology were not affected by feeding SB on weaning piglets [13]. While intensive studies focused on the period around weaning period, so far information on the role of SB in the health of neonatal piglets is limited.

In contrast to the established and stable microbiota of adult animals, the gut microbiota of neonates vary more among individuals and are less stable. The fragile ecological system of the neonatal gut is not only a disease risk to the newborn animal but also may have short- and long-term influence on the health later in life [5, 14, 15]. Thus, early intervention of the development of gut microbiota and mucosal immune system may have practical significance to improve the health of piglets.

During the early life of piglets, only a small quantity of butyrate is produced in the gut because of the immature microbiota and few fermentation substrates. Given the benefit role of butyrate in the gut health of weaning and growing piglets, we hypothesized that early intervention with additional butyrate can also impact the gut microbiota and immune system of the newborn piglets. In addition, the effects may last for the whole suckling period. Therefore, the aim of this study was to investigate the effects of early intervention with SB through oral administration on gut microbiota and the expression of inflammatory cytokines in piglets during the suckling period.

## Materials and Methods

### Ethics statement

The experiment was approved and conducted under the supervision of the Animal Care and Use Committee of Nanjing Agricultural University (Nanjing, Jiangsu province, China). All animal care procedures throughout the study followed Experimental Animal Care and Use Guidelines of China [16].

### Piglet experimental design

Ten litters of healthy neonatal piglets (10–11 piglets in each litter) derived from ten sows with the similar parity (3–4 parities) in a commercial maternal line herd (Duroc × Landrace × Yokshire) were randomly allocated to either the SB group or the control (CO) group. Each group consisted of five replicates (litters). From the age of 1 to 7 days, piglets in the SB group were orally administered with 7 to 13 ml sodium butyrate solution (pH = 7.4, 150 mmol/l) per day, respectively (each half of dose was given at 9:00 am and 3:00 pm). Piglets in the CO group were orally administered with the same dose of physiological saline. The solution was infused into the piglet mouth by an injector without the needle, piglets were gently put on the nursing pen immediately after the swallowing to avoid stress caused by the operation. All piglets were weaned on day 21. During the suckling period, piglets had free access to water, while no creep feed was provided. All piglets kept healthy during the experimental period, and there was no difference in the growth performance between CO and SB groups by recording the weight of piglets on days 1, 7, 14 and 21.

### Sampling

On days 8 and 21, one piglet from each litter was randomly selected and euthanized. The digesta in the stomach, distal ileum, and proximal colon were collected, and stored at -28°C for the analysis of microbial structure and butyrate concentration. To determine the expression of inflammatory-related genes of the ileum, the luminal fluid was drained, then distal segments of ileum (3–4 cm) were excised, washed with sterile phosphate buffer solution (PBS, pH 7.0), and immediately snap frozen in liquid nitrogen.

### Butyrate acid concentration analysis

The butyrate concentration in the stomach, ileum and colon was analyzed by using a capillary column gas chromatograph (GC-14B, Shimadzu, Japan; Capillary Column: 30 m × 0.32 mm × 0.25 μm film thickness) according to the description of a previous study [17].

### DNA Extraction, PCR amplification and Illumina MiSeq sequencing

The total genomic DNA was isolated from the digesta of stomach, ileum, and colon using a commercially available stool DNA extraction kit according to the manufacturer’s instructions (QIAamp DNA Stool Mini Kit, Qiagen, Hilden, Germany). The concentration of extracted DNA was determined by using a Nano-Drop 1000 spectrophotometer (Thermo Scientific Inc., Wilmington, DE, USA). The V4-V5 region of the bacterial *16S rRNA* gene was amplified by polymerase chain reaction (PCR) using bacterial universal primers 515F and 907R according to the description of previous studies [18, 19]. Purified amplicons were pooled in equimolar and paired-end sequenced (2 × 250) on an Illumina MiSeq platform according to the standard protocols at the Majorbio Bio-Pharm Technology (Shanghai, China).

### Bioinformatics analysis

Raw fastq files were demultiplexed and quality-filtered using QIIME (version 1.17) with the following criteria: the 250 bp reads were truncated at any site receiving an average quality score < 20 over a 10 bp sliding window, discarding the truncated reads that were shorter than 50 bp; exact barcode matching, 2 nucleotide mismatch in primer matching, reads containing ambiguous characters were removed; and only sequences that overlap longer than 10 bp were assembled according to their overlap sequence.

Operational taxonomic units (OTUs) were clustered with 97% similarity cutoff using UPARSE (version 7.1 http://drive5.com/uparse/), and chimeric sequences were identified and removed using UCHIME. To assess bacterial diversity among samples in a comparable manner, a randomly selected, 16274-sequence (the lowest number of sequences in the 60 samples) subset from each sample was compared for the phylogenetic affiliation by RDP Classifier (http://rdp.cme.msu.edu/) against the Silva (SSU115) 16S rRNA database using a confidence threshold of 70% [20]. We also calculated the coverage percentage using Good’s method [21], the abundance-based coverage estimator (ACE), the bias-corrected Chao richness estimator, and the Shannon and Simpson diversity indices using the MOTHUR program (http://www.mothur.org) [22]. Genera (OTUs) with relative abundances higher than 0.05% within total bacteria were defined as predominant genera (OTUs), and sorted for comparing the difference among different groups. As described by previous study [23], non-metric multidimensional scaling (NMDS) was employed to visualize relationships between samples by two-dimensional ordination plotting. The raw pyrosequencing reads were submitted to Sequencing Read Archive (SRA) database under the accession id: SRP074353.

### Real-time PCR quantification of total bacteria

Primer set Bact1369/Prok1492 was used for the quantification of total bacteria on an Applied Biosystems 7300 Real-Time PCR System (Applied Biosystems, USA) using SybrGreen as the fluorescent dye [24]. The PCR was performed according to the description of a previous study [25].

### RNA extraction, cDNA synthesis and real-time RT-PCR for gene expression of inflammatory cytokines

Total RNA of the ileum was isolated using TRIzol (Invitrogen, China), and quantified using a NanoDrop 1000 spectrophotometer (Thermo Scientific Inc., Wilmington, DE, USA). The absorption ratio (260:280 nm) of all the samples was between 1.8 and 2.0, which indicate a high purity of the RNA. One microgram RNA was reverse transcribed with standard reagents (Biocolors, China). The primers for inflammatory cytokines (*IL-6*, *IL-8*, *IL-1β*, *IL-18*, *TNF-α*, and *IFN-γ*), anti-inflammatory (*IL-10* and *TGF-β*), *histone deacetylase 1* (*HDAC1*), and housekeeping (*β-actin*, *GAPDH*, and *18S rRNA*) genes were listed in S1 Table [26–33]. The target genes and housekeeping genes were measured by quantitative real-time PCR with SybrGreen (Roche, Switzerland) and fluorescence was detected on an ABI 7300 sequence detector. The reaction system was 10 μl including 5 μl SYBR, 1 μl DNA (100 ng/μl), 0.5 μl forward and reserve primers (10 mmol/μl) and 3 μl double distilled water. Samples were incubated in the ABI 7300 sequence detector for an initial denaturation at 95°C for 10 min, followed by 35 PCR cycles of 95°C for 15 s, 60°C for 1 min and 72°C for 1 min. Of the three candidate housekeeping genes, *β-actin* was finally used for the accurate normalization by NormFinder software as described by Andersen et al. [34]. The expression of the genes was calculated relative to the expression of *β-actin* with formula 2^-ΔΔCt^. Amplification of specific transcripts was confirmed by melting curve profiles at the end of each PCR.

### Statistical analysis

Data were analyzed by SPSS 17.0 as a randomized block design, considering the SB treatment as the main effect and the replicate as a block. Litter was used as the experimental unit (n = 5) of all analysis, the individual pig represented the litter mean because one pig per litter was sampled on each day. The microbial data were analyzed by using the non-parametric Mann–Whitney test for independent samples. The data of inflammatory cytokines and butyrate concentration were evaluated by Student’s *t* test. Data were presented as group mean ± SD, significant differences were declared when *P* < 0.05. The correlations between the expression of inflammatory cytokines and bacterial community compositions were assessed by Pearson’s correlation test using Graphpad Prism version 5.00 (Graphpad Software, San Diego, CA, USA).

## Results

### Gut butyrate concentration

As shown in S2 Table, SB had no significantly effect on the butyrate concentration in the stomach, ileum, and colon of piglets on days 8 and 21.

### Gut microbial community

**Fig 1:**
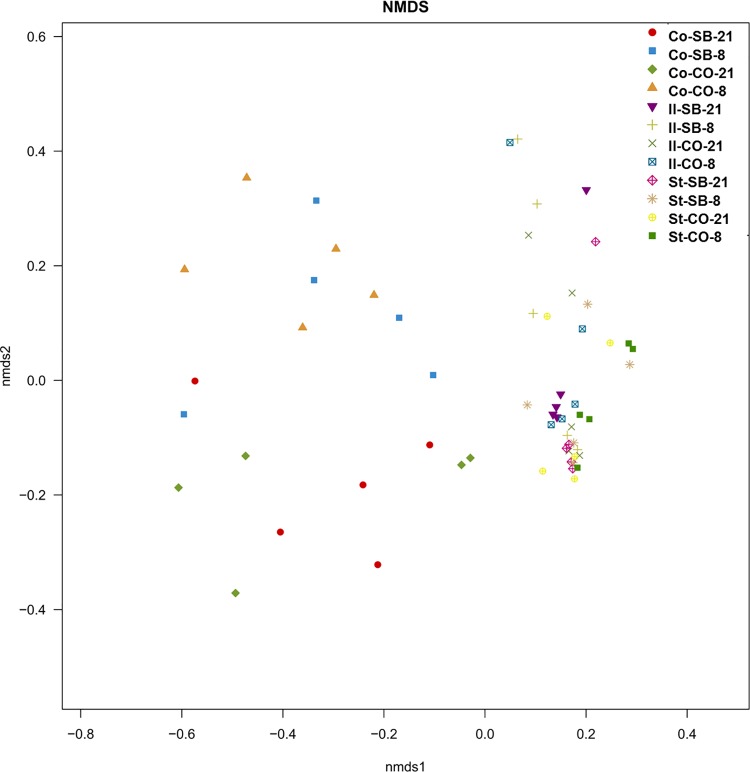
Nonmetric multidimensional scaling analysis (NMDS). NMDS representation of OTU-based clustering (0.03 genetic distance) of data from the V4-V5 region of the bacterial *16S rRNA* gene. Counts of each OTU within each piglet were standardized to percentage, square-root transformed and a Bray-Curtis similarity matrix was calculated.

Across all 60 samples, 1,873,852 quality sequences were classified as bacteria with a read length higher than 250 bp. The average length of the quality sequences was 445.12 bp. The rarefaction curves generated by MOTHUR plotting the number of reads by the number of OTUs tended to approach the saturation plateau (S1 Fig). The statistical estimates of species richness for 16274-sequence subsets from each sample at a genetic distance of 3% are presented in Table 1. In the stomach, the richness estimators (ACE and Chao) in the SB group were significantly higher than in the CO group on day 8 (*P* < 0.05), while the diversity indices (Shannon and Simpson) were not affected by the SB treatment. SB significantly decreased the ACE value of ileal microbiota (*P* < 0.05), while there was no difference in diversity indices between SB and CO groups. As compared to the CO group, the richness estimators Chao and diversity of colonic microbiota in the SB group significantly increased at the age of 8 days (*P* < 0.05); the addition of SB did not affect the richness estimators and diversity index in comparison with the control on day 21. Non-metric multidimensional scaling (NMDS) of Bray-Curtis similarity matrices for OTU-based clustering indicated that the composition of bacterial communities in the samples was separated by the three gut segments (Fig 1). However, samples from the stomach, ileum, and colon of piglets on days 8 and 21 could not be separated by the SB treatment.

**Table 1:** Diversity estimation of the *16S rRNA* gene libraries from microbiota in the stomach, ileum, and colon of piglets in the sodium butyrate (SB) and control (CO) groups (n = 5).

Item	8 d		21 d	
	CO	SB	CO	SB
**Stomach**				
**Ace**	209.46±16.79	298.88±32.11*	322.83±37.52	317.08±38.73
**Chao**	200.99±14.33	284.39±21.91*	297.11±40.32	287.91±14.19
**Shannon**	1.75± 0.16	2.19± 0.25	2.00± 0.40	1.95± 0.20
**Simpson**	0.30± 0.05	0.24± 0.05	0.31± 0.07	0.30± 0.05
**Ileum**				
**Ace**	326.86±22.53	251.78±12.80*	239.96±32.38	281.18±11.16
**Chao**	293.92±22.45	239.22±13.03	222.67±42.23	248.36± 9.23
**Shannon**	2.08± 0.17	2.19± 0.28	2.17± 0.44	2.21± 0.15
**Simpson**	0.26± 0.05	0.23± 0.05	0.26± 0.06	0.24± 0.05
**Colon**				
**Ace**	199.82±21.35	253.93±19.30	271.73±14.88	267.88±16.83
**Chao**	194.78±15.15	267.79±13.67**	279.57±16.44	270.74±17.08
**Shannon**	2.78± 0.17	3.47± 0.12**	2.79± 0.33	2.93± 0.34
**Simpson**	0.16± 0.03	0.06± 0.01*	0.20± 0.09	0.15± 0.05

* means the significantly difference (*P* < 0.05) between SB and CO groups

** means the significantly difference (*P* < 0.01) between SB and CO groups, the same as follows.

At the phylum level, Firmicutes was the most predominant phylum in the stomach and ileum (Table 2) of piglets on days 8 and 21. In the stomach, the relative abundance of Firmicutes in the SB group was significantly lower than the CO group, whereas the abundance of Proteobacteria and Actinobacteria in the SB group was significantly higher than the CO group on day 8 (*P* < 0.05). In the colon, SB significantly decreased the abundance of Bacteroidetes, and increased the abundance of Firmicutes and Actinobacteria (*P* < 0.05) on day 8 (Table 2).

**Table 2:** Relative abundance of microbial phylum (percentage) in the stomach, ileum, and colon of piglets in the sodium butyrate (SB) and control (CO) groups (n = 5)^1^.

phylum	8d		21d	
	CO	SB	CO	SB
**Stomach**				
Firmicutes	98.114±0.351	92.400±3.848**	93.615±3.848	97.601±0.104
Proteobacteria	0.678±0.127	3.105±2.178*	3.284±2.178	1.006±0.227
Bacteroidetes	0.604±0.107	1.996±0.963	1.891±0.963	0.680±0.122
Actinobacteria	0.274±0.107	1.314±0.346*	0.579±0.346	0.524±0.222
Fusobacteria	0.223±0.090	0.849±0.294	0.462±0.294	0.127±0.018
Candidate_division_TM7	0.096±0.059	0.295±0.033	0.089±0.033	0.045±0.026
**Ileum**				
Firmicutes	88.655±6.828	80.176±9.034	93.397±3.129	95.441±1.711
Proteobacteria	5.766±4.598	15.205±7.793	1.140±0.405	0.857±0.504
Fusobacteria	3.329±2.454	2.297±1.433	0.611±0.319	0.130±0.026
Actinobacteria	0.926±0.236	1.146±0.447	3.941±2.210	2.880±0.791
Bacteroidetes	0.784±0.216	0.642±0.244	0.067±0.021	0.071±0.021
Candidate_division_TM7	0.396±0.186	0.402±0.250	0.794±0.457	0.611±0.453
**Colon**				
Bacteroidetes	64.977±3.539	41.261±4.470**	19.042±6.006	29.486±6.262
Firmicutes	30.011±4.411	46.887±2.366**	75.092±7.608	67.743±6.866
Fusobacteria	2.274±1.230	7.628±5.487	0.220±0.131	0.466±0.252
Proteobacteria	1.049±0.589	3.009±1.705	0.918±0.442	1.243±0.892
Spirochaetae	0.987±0.981	0.018±0.017	0.036±0.033	0.020±0.013
Verrucomicrobia	0.401±0.242	0.852±0.800	0.000±0.000	0.004±0.002
Cyanobacteria	0.144±0.144	0.000±0.000	0.078±0.075	0.020±0.013
Actinobacteria	0.079±0.015	0.287±0.082*	2.378±1.439	0.631±0.163
Synergistetes	0.024±0.018	0.001±0.001	2.071±1.832	0.298±0.216
Tenericutes	0.001±0.001	0.023±0.023	0.154±0.102	0.098±0.086

^**1**^Phylum with relative abundances higher than 0.05% within total bacteria were sorted and showed in the table.

* means the significantly difference (*P* < 0.05) between SB group and CO group.

** means the significantly difference (*P* < 0.01) between SB group and CO group.

At the class level, SB significantly decreased the abundance of Bacilli, and increased Actinobacteria, Flavobacteriia and Betaproteobacteria (*P* < 0.05) in the stomach on day 8 (S3 Table). SB significantly decreased the abundance of Flavobacteriia (*P* < 0.05) in the ileum on day 8 (S4 Table). In the colon, a lower abundance of Bacteroidia and a higher abundance of Negativicutes and Actinobacteria (*P* < 0.05) were found in the SB group on day 8 as compared with the CO group (S5 Table).

Family-level analysis revealed that Lactobacillaceae in the stomach was significantly decreased in the relative abundance by the SB treatment, whereas Pasteurellaceae, Flavobacteriaeae, Micrococcaceae and Aerococcaceae were increased (*P* < 0.05) on day 8 (S6 Table), Flavobacteriaeae in the ileum (*P* < 0.05) was increased by the SB treatment (S7 Table).

Genus-level analysis revealed that the genera *Lactobacillus*, *Streptococcus*, and *Veillonella* were the three predominant genera in the stomach and ileum of piglets on days 8 and 21. *Bacteroides* and *Lactobacillus* were the predominant genera in the colon. As shown in Table 3, genera *Acinetobacter*, *Actinobacillus*, *Facklamia*, *Globicatella*, *Kocuria*, *Rothia*, unclassified Leptotrichiaceae, unclassified Neisseriaceae, and unclassified Prevotellaceae in the stomach were significantly increased in relative abundance by the SB treatment, whereas the abundances of *Lactobacillus* decreased on day 8 (*P* < 0.05). In the ileum (S9 Table), SB only decreased the abundance of *Sarcina* at the age of 21 days (*P* < 0.05) and tended to increase the abundance of *Bergeyella* on day 8. SB significantly decreased the abundance of *Peptostreptococcus* in the colon on day 21 (*P* < 0.05). A higher tendency was observed in the relative abundance of genera *Corynebacterium*, *Faecalibacterium*, *Odoribacter*, *Roseburia*, *Subdoligranulum*, and unclassified Lachnospiraceae in the colon of piglets treated with SB on day 8 (S10 Table).

**Table 3:** Relative abundance of microbial genera (percentage) in the stomach of piglets in the sodium butyrate (SB) and control (CO) groups (n = 5)^1^.

Genus	8 d		21 d	
	CO	SB	CO	SB
*Lactobacillus*	95.154±0.922	83.759±5.204*	79.413±9.895	83.773±9.112
*Streptococcus*	0.951±0.307	4.125±2.229	1.081±0.414	5.403±4.246
*Clostridium*_*sensu*_*stricto*_1	0.464±0.335	0.527±0.065	0.878±0.453	0.626±0.345
*Moraxella*	0.271±0.068	1.060±0.390	0.869±0.593	0.395±0.181
*Veillonella*	0.217±0.131	1.339±0.826	0.326±0.126	2.593±2.377
*Actinobacillus*	0.181±0.047	0.866±0.323*	1.649±1.144	0.815±0.355
*Porphyromonas*	0.168±0.080	0.632±0.230	0.356±0.231	0.119±0.026
*Sarcina*	0.143±0.135	0.119±0.109	7.949±7.926	0.002±0.001
unclassified Lactobacillales	0.137±0.045	0.240±0.137	0.134±0.049	0.522±0.297
*Bacteroides*	0.132±0.034	0.053±0.027	0.179±0.094	0.074±0.039
*Fusobacterium*	0.126±0.060	0.227±0.071	0.174±0.090	0.082±0.020
*Turicibacter*	0.098±0.048	0.169±0.043	0.161±0.096	0.165±0.066
norank Candidate_division_TM7	0.096±0.059	0.295±0.134	0.089±0.033	0.051±0.021
*Rothia*	0.079±0.029	0.515±0.214*	0.124±0.046	0.426±0.185
*Corynebacterium*	0.078±0.039	0.426±0.200	0.206±0.163	0.794±0.658
*Acinetobacter*	0.004±0.002	0.154±0.129*	0.003±0.001	0.012±0.007
*Actinomyces*	0.057±0.050	0.037±0.016	0.159±0.121	0.034±0.012
*Aerococcus*	0.011±0.005	0.161±0.094	0.125±0.109	0.249±0.214
*Alloprevotella*	0.049±0.008	0.194±0.060	0.312±0.134	0.164±0.074
*Arcanobacterium*	0.015±0.006	0.054±0.025	0.009±0.004	0.017±0.006
*Arthrobacter*	0.001±0.001	0.052±0.028	0.018±0.017	0.005±0.004
*Atopostipes*	0.002±0.001	0.011±0.009	0.072±0.071	0.001±0.001
*Bergeyella*	0.066±0.016	0.512±0.190	0.402±0.267	0.089±0.041
*Chryseobacterium*	0.011±0.008	0.244±0.204	0.011±0.007	0.117±0.085
*Enterococcus*	0.016±0.016	0.063±0.046	0.006±0.003	0.046±0.043
*Facklamia*	0.000±0.000	0.055±0.031**	0.023±0.015	0.073±0.041
*Fastidiosipila*	0.000±0.000	0.005±0.003	0.068±0.054	0.001±0.001
*Gemella*	0.020±0.008	0.079±0.024	0.016±0.005	0.030±0.021
*Globicatella*	0.022±0.006	0.080±0.023*	0.064±0.044	0.129±0.056
*Haemophilus*	0.055±0.019	0.406±0.188	0.431±0.276	0.144±0.022
*Helcococcus*	0.030±0.014	0.027±0.019	0.076±0.061	0.040±0.019
*Howardella*	0.009±0.004	0.013±0.010	0.020±0.007	0.074±0.063
*Ignavigranum*	0.004±0.004	0.006±0.002	0.138±0.138	0.007±0.004
*Jeotgalicoccus*	0.014±0.011	0.017±0.007	0.080±0.077	0.025±0.009
*Kocuria*	0.000±0.000	0.085±0.055**	0.011±0.004	0.074±0.047
*Leptotrichia*	0.052±0.016	0.453±0.394	0.194±0.136	0.086±0.042
*Neisseria*	0.006±0.006	0.009±0.004	0.086±0.071	0.042±0.014
norank Bacteroidales S24-7	0.024±0.014	0.026±0.009	0.114±0.057	0.026±0.013
*Nosocomiicoccus*	0.004±0.004	0.004±0.003	0.223±0.218	0.009±0.005
*Pasteurella*	0.020±0.007	0.144±0.003	0.001±0.001	0.001±0.001
*Peptostreptococcus*	0.034±0.009	0.080±0.043	0.066±0.036	0.054±0.023
*Prevotella*	0.030±0.012	0.010±0.003	0.056±0.036	0.022±0.010
*Proteocatella*	0.014±0.008	0.086±0.047	0.056±0.028	0.030±0.010
*Psychrobacter*	0.003±0.001	0.061±0.040	0.006±0.005	0.008±0.004
uncultured Prevotellaceae	0.022±0.007	0.014±0.006	0.078±0.066	0.014±0.005
uncultured Ruminococcaceae	0.042±0.010	0.191±0.093	0.178±0.085	0.048±0.016
*Weissella*	0.001±0.001	0.086±0.041	0.038±0.024	0.438v0.410
unclassified Lachnospiraceae	0.032±0.009	0.065±0.026	0.175±0.082	0.073±0.022
unclassified Leptotrichiaceae	0.044±0.018	0.169±0.054*	0.094±0.072	0.027±0.019
unclassified Moraxellaceae	0.028±0.011	0.156±0.106	0.014±0.003	0.023±0.013
unclassified Neisseriaceae	0.016±0.007	0.054±0.017*	0.046±0.025	0.042±0.031
unclassified Porphyromonadaceae	0.022±0.007	0.097±0.041	0.114±0.079	0.045±0.016
unclassified Prevotellaceae	0.023±0.005	0.147±0.050*	0.204±0.105	0.063±0.015

^**1**^Genera with relative abundances higher than 0.05% within total bacteria were sorted and showed in the table.

* means the significantly difference (P < 0.05) between SB group and CO group.

** means the significantly difference (P < 0.01) between SB group and CO group

At the OTU level, SB significantly increased the relative abundance of *Actinobacillus porcinus*-, *Rothia*-, *Actinobacillus minor*-, *Kocuria carniphila*-, *Corynebacterium*-, Leptotrichiaceae-, and *Actinomyces-*related OTUs (*P* < 0.05) in the stomach on day 8 (Table 4). In the ileum (S11 Table), the relative abundance of Erysipelotrichaceae-related OTU of piglets supplemented with SB was higher than that from the CO group at the age of 8 days (*P* < 0.05). On day 21, SB significantly increased the relative abundance of Peptostreptococcaceae- and Lactobacillales-related OTUs, and decreased the relative abundance of *Sarcina*-related OTU (*P* < 0.05). In the colon (S12 Table), SB treatment significantly increased the relative abundance of *Prevotella* sp.-, *Bacteroides*-, and Ruminococcaceae-related OTUs on day 8, and decreased the relative abundance of *Peptostreptococcus*-related OTU on day 21.

**Table 4:** Relative abundance of microbial OTUs (percentage) in the stomach of piglets in the sodium butyrate (SB) and control (CO) groups (n = 5)^1^.

OTUName	8 d		21 d		Annotation^2^
	CO	SB	CO	SB	
OTU655	12.88±6.898	25.90±14.85	19.91±9.958	51.86±4.510	g__*Lactobacillus*
OTU792	24.69±2.643	24.95±3.902	11.82±2.184	22.47±1.509	g__*Lactobacillus*
OTU13	19.68±4.168	3.557±1.070	7.670±3.800	3.250±0.600	g__*Lactobacillus*
OTU379	0.149±0.035	0.496±0.169	0.345±0.139	0.753±0.281	s__*Streptococcus*_*gallolyticus*_subsp._*macedonicus*
OTU527	4.943±0.453	0.771±0.176	4.027±0.984	5.592±0.238	g__*Lactobacillus*
OTU535	3.966±0.341	0.688±0.176	3.216±0.746	3.988±0.243	g__*Lactobacillus*
OTU581	0.139±0.069	1.038±0.591	0.367±0.129	0.198±0.087	g__*Veillonella*
OTU806	0.087±0.041	0.287±0.194	2.176±1.661	2.352±1.118	g__*Lactobacillus*
OTU842	0.427±0.184	0.320±0.251	4.441±0.144	0.938±0.242	g__*Lactobacillus*
OTU235	0.273±0.272	0.347±0.206	0.413±0.246	0.982±0.401	s__*Lactobacillus*_*coleohominis*
OTU301	0.092±0.033	0.544±0.254*	0.241±0.103	0.284±0.094	s__*Actinobacillus*_*porcinus*
OTU820	4.070±1.757	7.213±4.334	1.866±1.811	0.268±0.230	s__*Lactobacillus*_*johnsonii*
OTU266	0.286±0.203	0.336±0.082	0.183±0.089	0.202±0.047	g__*Clostridium*_sensu_stricto_1
OTU616	0.001±0.001	0.086±0.041	0.047±0.028	0.548±0.510	s__*Weissella*_*paramesenteroides*
OTU431	0.158±0.095	0.291±0.070	0.151±0.070	0.229±0.069	f__Peptostreptococcaceae
OTU145	0.082±0.034	0.141±0.098	0.092±0.038	0.122±0.055	o__Lactobacillales
OTU47	0.078±0.028	0.512±0.212*	0.146±0.049	0.254±0.103	g__*Rothia*
OTU622	0.007±0.003	0.118±0.089	0.040±0.022	0.481±0.406	s__*Corynebacterium*_*testudinoris*
OTU457	0.271±0.068	1.053±0.384	0.239±0.124	0.177±0.066	g__*Moraxella*
OTU249	0.017±0.007	0.241±0.207	0.165±0.070	0.413±0.160	g__*Lactobacillus*
OTU316	0.011±0.005	0.371±0.094	0.076±0.038	0.308±0.162	g__*Lactobacillus*
OTU678	0.011±0.005	0.161±0.094	0.156±0.134	0.035±0.019	g__*Aerococcus*
OTU76	0.116±0.073	0.161±0.064	0.048±0.028	0.085±0.028	s__[*Clostridium*]_*glycolicum*
OTU573	0.216±0.035	1.062±0.388	0.259±0.064	0.240±0.079	g__*Streptococcus*
OTU699	0.034±0.022	0.105±0.051	0.031±0.012	0.201±0.143	s__*Streptococcus*_*orisratti*
OTU315	0.013±0.005	0.143±0.098	0.085±0.034	0.195±0.121	g__*Lactobacillus*
OTU303	1.483±0.561	0.882±0.445	0.364±0.274	0.364±0.274	g__*Lactobacillus*
OTU317	0.011±0.004	0.094±0.055	0.074±0.037	0.184±0.117	g__*Lactobacillus*
OTU551	0.069±0.047	0.046±0.039	0.218±0.103	0.219±0.063	s__*Lactobacillus*_*mucosae*
OTU441	0.098±0.048	0.169±0.043	0.161±0.096	0.165±0.066	g__*Turicibacter*
OTU46	0.046±0.018	0.146±0.082	0.111±0.037	0.179±0.046	s__*Actinobacillus*_*rossii*
OTU462	0.046±0.008	0.174±0.064	0.271±0.133	0.148±0.078	s__*Prevotella*_sp._canine_oral_taxon_282
OTU722	0.055±0.019	0.406±0.189	0.158±0.059	0.162±0.015	g__*Haemophilus*
OTU664	0.001±0.001	0.081±0.072	0.023±0.022	0.134±0.123	g__*Corynebacterium*
OTU310	0.043±0.011	0.175±0.039*	0.350±0.206	0.133±0.059	g__*Actinobacillus*
OTU709	0.011±0.006	0.041±0.019	0.029±0.010	0.128±0.093	g__*Lactobacillus*
OTU252	0.104±0.094	0.061±0.023	0.018±0.001	0.124±0.091	g__*Clostridium*_sensu_stricto_1
OTU424	0.218±0.071	1.302±0.775	0.069±0.029	0.120±0.074	g__Streptococcus
OTU120	1.285±0.463	0.537±0.222	0.210±0.112	0.126±0.068	g__*Lactobacillus*
OTU386	0.001±0.001	0.034±0.026	0.013±0.010	0.120±0.116	s__*Lactobacillus*_*amylotrophicus*
OTU125	0.010±0.005	0.104±0.075	0.031±0.029	0.118±0.075	s__*Corynebacterium*_*freneyi*
OTU763	0.049±0.014	0.081±0.043	0.031±0.006	0.087±0.032	o__Lactobacillales
OTU350	0.036±0.036	0.028±0.017	0.011±0.010	0.099±0.092	g__*Corynebacterium*
OTU187	0.105±0.038	0.539±0.224	0.148±0.091	0.076±0.025	g__*Porphyromonas*
OTU302	0.000±0.000	0.085±0.055**	0.014±0.004	0.092±0.056	s__*Kocuria*_*carniphila*
OTU217	0.009±0.004	0.013±0.010	0.020±0.007	0.074±0.063	g__*Howardella*
OTU158	0.001±0.001	0.153±0.126	0.010±0.006	0.086±0.066	g__*Chryseobacterium*
OTU239	0.001±0.001	0.040±0.020**	0.011±0.009	0.086±0.073	g__*Corynebacterium*
OTU474	0.019±0.005	0.064±0.023	0.031±0.018	0.067±0.030	s__*Globicatella*_sp._canine_oral_taxon_218
OTU202	0.094±0.042	0.214±0.072	0.136±0.070	0.066±0.016	g__*Fusobacterium*
OTU579	0.000±0.000	0.001±0.001	0.000±0.000	0.076±0.049	s__*Lactobacillus*_agilis
OTU532	0.064±0.038	0.016±0.007	0.236±0.220	0.058±0.033	g__*Lactobacillus*
OTU667	0.001±0.001	0.012±0.010	0.035±0.034	0.072±0.034	g__*Globicatella*
OTU92	0.001±0.001	0.002±0.001	0.028±0.012	0.057±0.035	g__*Streptococcus*
OTU818	0.806±0.360	1.026±0.728	0.141±0.102	0.054±0.034	g__*Lactobacillus*
OTU839	0.004±0.002	0.029±0.015	0.015±0.007	0.052±0.085	g__*Veillonella*
OTU22	0.066±0.016	0.512±0.190	0.117±0.075	0.050±0.016	s__*Bergeyella*_*zoohelcum*
OTU625	0.034±0.009	0.080±0.043	0.066±0.036	0.048±0.023	g__*Peptostreptococcus*
OTU419	0.077±0.054	0.148±0.099	0.081±0.033	0.047±0.021	p__Candidate_division_TM7
OTU756	0.006±0.006	0.063±0.054	0.003±0.003	0.058±0.056	s__*Streptococcus*_*parauberis*
OTU10	0.004±0.004	0.341±0.334	0.036±0.008	0.045±0.024	g__*Leptotrichia*
OTU85	0.022±0.007	0.097±0.041	0.113±0.079	0.045±0.016	f__Porphyromonadaceae
OTU322	0.014±0.013	0.056±0.044	0.005±0.002	0.052±0.052	s__*Enterococcus*_*italicus*
OTU427	0.000±0.000	0.007±0.007	0.046±0.020	0.051±0.023	g__*Moraxella*
OTU26	0.000±0.000	0.000±0.000	0.093±0.023	0.048±0.031	s__*Bergeyella*_*zoohelcum*
OTU558	0.025±0.010	0.175±0.079	0.021±0.005	0.037±0.012	s__*Streptococcus*_*thoraltensis*_DSM_12221
OTU611	0.048±0.043	0.078±0.034	0.084±0.033	0.032±0.010	g__*Porphyromonas*
OTU692	0.020±0.008	0.079±0.024	0.016±0.005	0.030±0.021	g__*Gemella*
OTU508	0.014±0.008	0.086±0.047	0.056±0.028	0.030±0.021	s__*Frigovirgula*_sp._canine_oral_taxon_058
OTU602	0.019±0.004	0.136±0.050	0.039±0.009	0.030±0.008	f__Prevotellaceae
OTU700	0.048±0.015	0.111±0.063	0.124±0.101	0.030±0.017	g__*Leptotrichia*
OTU229	0.019±0.006	0.091±0.048	0.015±0.002	0.033±0.013	s__*Streptococcus*_*pluranimalium*
OTU451	0.001±0.001	0.007±0.007	0.137±0.079	0.026±0.012	f__Prevotellaceae
OTU184	0.000±0.000	0.001±0.001	0.109±0.073	0.025±0.011	g__*Bacteroides*
OTU101	0.024±0.011	0.128±0.085	0.010±0.003	0.020±0.014	f__Moraxellaceae
OTU68	0.004±0.002	0.296±0.277	0.018±0.012	0.024±0.015	s__*Lactobacillus*_sp._KC45b
OTU32	0.000±0.000	0.079±0.076	0.429±0.427	0.021±0.021	s__*Clostridium*_sp._ND2
OTU254	0.018±0.005	0.129±0.056**	0.097±0.078	0.020±0.015	f__Leptotrichiaceae
OTU860	0.011±0.010	0.004±0.004	0.077±0.074	0.018±0.010	s__*Jeotgalicoccus*_sp._M3T9B12
OTU96	0.019±0.006	0.060±0.033	0.008±0.007	0.015±0.008	g__*Lactobacillus*
OTU439	0.003±0.001	0.029±0.011*	0.121±0.100	0.010±0.002	g__*Actinomyces*
OTU178	0.004±0.004	0.004±0.003	0.279±0.271	0.011±0.006	g__*Nosocomiicoccus*

^**1**^OTUs with relative abundances higher than 0.05% within total bacteria were sorted and showed in the table.

^2^The consensus sequence of each OTU was annotated to the closest lineage using MOTHUR program against the SILVA 16S rRNA reference database. s = species; g = genus; f = family; o = order.

* means the significantly difference (P < 0.05) between SB group and CO group.

** means the significantly difference (P < 0.01) between SB group and CO group.

Because MiSeq sequencing analysis can only reflect the relative abundance of bacteria, quantitative real-time PCR was used to determine the completed *16S rRNA* gene copies of bacteria in the stomach, ileum, and colon of piglets. As shown in S2 Fig, SB treatment had no effect on the total numbers of bacteria in the stomach, ileum, and colon of piglets on days 8 and 21.

### Gene expression of inflammatory cytokines

**Fig 2:**
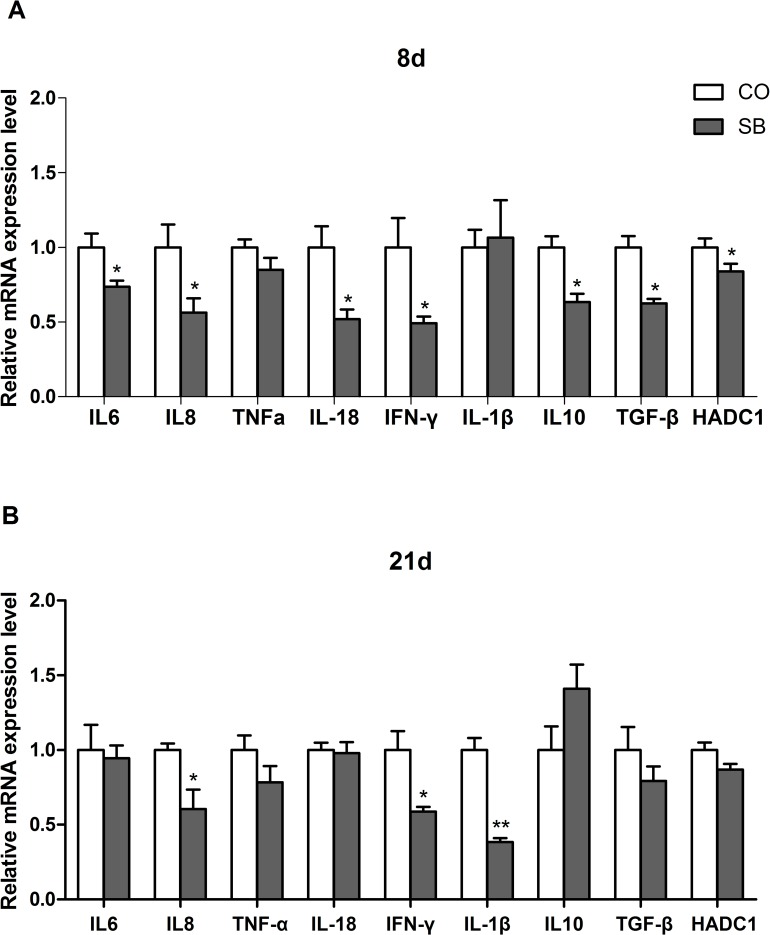
Gene expression of inflammatory cytokines. The relative gene expression of inflammatory cytokines in the ileum of piglets in the sodium butyrate (SB) and control (CO) groups. The values were calculated relative to the expression of β-actin with formula 2-ΔΔCt.

On day 8, SB treatment significantly down-regulated the expression of pro-inflammatory genes *IL-6*, *IL-8*, and *IFN-γ*, and anti-inflammatory genes *IL-10* and *TGF-β* in the ileum of piglets (*P* < 0.05). There was no difference in the expression of genes *TNF-α* and *IL-1β* between two groups. The expression of the *HDAC1* gene in the SB group was lower than the control group (Fig 2A). On day 21, SB significantly down-regulated the expression of pro-inflammatory genes *IL-8*, *IFN-γ*, and *IL-1β* in the ileum of piglets (*P* < 0.05). No difference in the expression of the pro-inflammatory genes (*IL-6*, *TNF-α*, and *IL-18*) and anti-inflammatory genes (*IL-10* and *TGF-β*) was observed between two groups. SB had no effect on the expression of gene *HDAC1* at the age of 21 days (Fig 2B)**.**

### Correlation between the microbial composition and inflammatory cytokine expression

**Fig 3:**
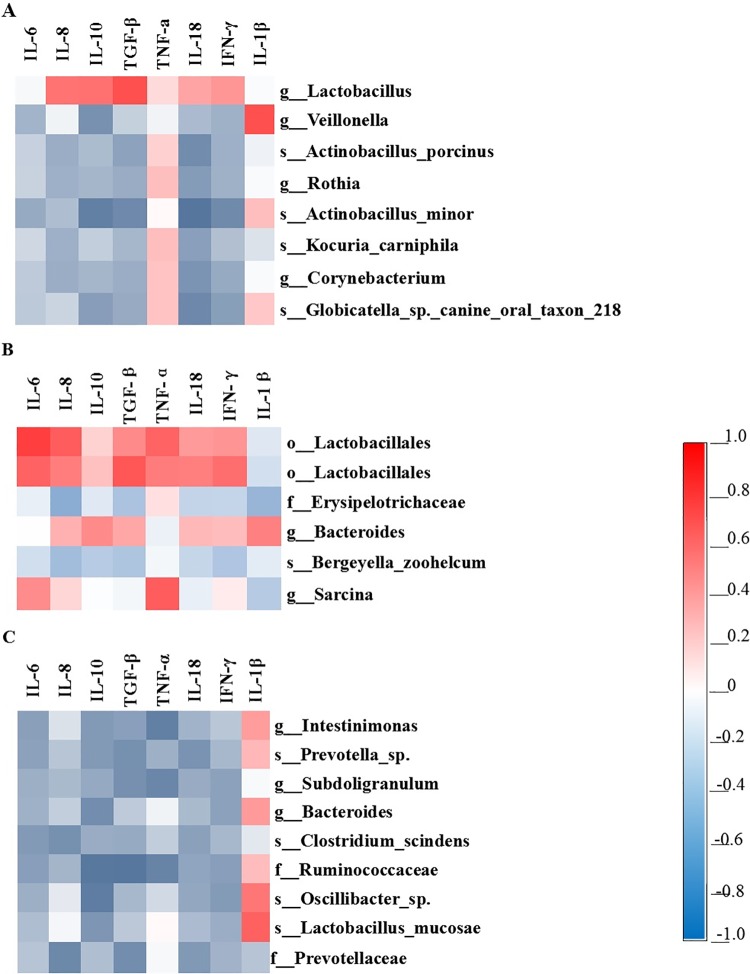
Correlation analysis between microbiota composition and inflammatory cytokines. Correlation analysis between the relative abundance of microbiota (at the OTU level) in the stomach (A), ileum (B), and colon (C) affected by the sodium butyrate treatment and the gene expression of inflammatory cytokines in the ileum of piglets. The color is according to the Pearson correlation coefficient distribution; red presents significant positive correlation, blue represents significantly negative correlation.

A Pearson’s correlation analysis was carried out to determine the relationships between microbial composition and inflammatory cytokine expression. Fig 3A showed that the relative mRNA expression of genes *IL-10*, *TGF-β*, *IL-18*, and *IFN-γ* was negatively correlated with the abundance of *Actinobacillus minor*-related OTU in the stomach. The abundance of *Lactobacillus*-related OTU was positively correlated with the expression of *TGF-β*. The abundance of *Veillonella-*related OTU was positively correlated with the expression of *IL-1β*. The abundance of *Globicatella*-related OTU was negatively correlated with the expression of *IL-18*. As shown in Fig 3B, the relative mRNA expression of *IL-6*, *IL-8*, and *TGF-β* was positively correlated with the Lactobacillales-related OTU in the ileum. The abundance of *Sarcina* was positively correlated with the expression of *TNF-α*, while the expression of *IL-8* was negatively correlated with the abundance of Erysipelotrichaceae. Fig 3C shows that the expression of *IL-8* was negatively correlated with the abundance of Prevotellaceae-related OTU in the colon. The expression of *IL-10* was negatively correlated with the abundance of Ruminococcaceae- and *Oscillibacter* sp.-related OTUs. The expression of *TGF-β* was negatively correlated with the abundance of Ruminococcaceae-related OTU. The expression of *TNF-α* was negatively correlated with the abundance of *Intestinimonas*-, *Subdoligranulum*-, and Ruminococcaceae-related OTUs.

## Discussion

Increasing evidence has demonstrated that early appropriate microbiota colonization could change the pattern of microbial composition as well as immunological maturation [35, 36]. In the present study, we investigated the effects of early intervention with SB on gut microbial composition and the expression of inflammatory cytokine in neonatal piglets. We found that SB treatment significantly increased the diversity of the stomach microbiota, and affected the gene expression of inflammatory cytokines in the ileum, but had low impact on the intestinal bacterial community.

Most studies today focus on the gut microbiota in the post-weaning pigs or growing pigs, whereas research on the effect of SB on microbial composition in neonatal piglets is limited. This is the first report of effects of sodium butyrate on gut microbiota in neonatal piglets using deep-sequencing methods. Previous study showed that SB supplement in the diet reduced the coliform count and increased the counts of *Lactobacillus* spp. in the ileum [12]. However, the present study revealed that oral administration of SB had no impact on the number of total bacteria as well as the abundance of most genera, both in the stomach, ileum, and colon. This is also inconsistent with the findings of a previous study where the butyrate perturbation had significant effect on microbial composition of rumen [37]. The inconsistent results may be due to the different methods for SB supplement or different animal models used in different studies.

SB had no effect on the numbers of total bacteria, while increased the richness estimators (ACE and Chao) of stomach, decreased the richness estimator (ACE) of ileum and significantly increased the richness estimator (Chao) and the diversity of microbiota in the colon on day 8, respectively, which means that the incoherent effects of SB may be primarily associated with the different segment of intestine. The findings are in line with a previous study where dietary SB decreased the ileal microbial diversity whereas increased the diversity in the colon of weaned piglets [38], which suggests that dietary SB may be benefit for the development of hindgut microbiota in piglets. At the age of 21 days, however, oral administration of SB had no effect on the diversity or composition of gut microbiota, which suggests that early intervention with SB may not have a long-term effect on gut microbiota in piglets.

The phyla Firmicutes and Bacteroidetes are known for a fermentative metabolism and degradation of polysaccharide, oligosaccharides, protein and amino acid [39, 40]. In the current study, SB decreased the abundance of Firmicutes and tended to increase the abundance of Bacteroidetes in the stomach, whereas the reverse result was observed in the colon, which suggests that the role of SB in modulating microbes is specific to different bacterial groups and gut segments.

At the genus level, our study revealed the most predominant genus in the stomach to be classified as *Lactobacillus* at the age of 8 and 21 days, which is in consistent with a previous study [41]. It is well known that *Lactobacillus* has the properties including anti-inflammatory and anti-bacterial activities. A previous study demonstrated that *Lactobacillus* plays a protective role by producing compounds such as hydrogen peroxide (H_2_O_2_) and lactic acid which inhibit the growth of potential pathogens [41]. In this study, SB significantly decreased the abundance of *Lactobacillus* on day 8 in the stomach and ileum, which may not play negative role in the gut health since the microbial diversity was increased by SB treatment, and *Lactobacillus* was still the most predominant genus. Unlike the stomach, SB had very low impact on the microbial composition both in ileum and colon, the possible reason is that SB may be partly absorbed by the stomach [42], in addition, the individual variation in response to SB treatment was found, and the low replicates (five) used in this study may also impair the statistical significance. Of note, SB significantly increased the relative abundance of *Prevotella* sp. in the colon, which is in accordance with the previous study where a positive correlation between *Prevotella* spp. and butyrate was found[43].

In recent years, butyrate has become a promising agent to treat colonic inflammation due to its capacities of antibacterial [44] and anti-inflammatory [11, 45]. It was reported that butyrate decreased the expression of pro-inflammatory cytokine mRNA in Crohn's disease patients. Chang et al. performed assay in the bone marrow-derived macrophages and found that butyrate decreased the pro-inflammatory cytokines expression through inhibition of histone deacetylases [45]. As showed by Miseq sequencing, SB had no effect on the bacterial composition in the ileum, thus it is supposed that the role of butyrate in regulating immune is mainly because it can act on cells via inhibition of HDAC and controls the acetylation state of histones, and modulates the transcription of several genes. In the present study, we found that SB decreased the expression of most detected pro-inflammatory cytokines and HDAC1, which is in agreement with previous studies.

Zhang et al. found that butyrate at a 2 mmol/l decreased the expression of *IL-6* and *TNF-α* in cultured murine bone marrow-derived mast cells stimulated with TNP-BSA [46]. Also, butyrate decreased the expression level of *IL-6* in the colon organ cultures stimulated with dextran sulphate sodium [47]. Similarly, our in vivo study also found that oral administration of SB decreased the expression of pro-inflammatory cytokines *IL-6*, *IL-8*, *IL-18*, and *IFN-γ*, however, no change in TNF-α was found on days 8 and 21. Correlation analysis in the study showed that the expression of *IL-6* and *IL-8* was positively related to the Lactobacillales-related OTU in the ileum, however, previous studies found that dietary SB increased the count of *Lactobacillus* and inhibited the pathogens such as *Esccherichia coli*, which eventually plays benefit roles in maintaining the normal mucosal immunity [12, 48]. Our results indicate that the role of early intervention with SB in regulating immune is mainly via inhibiting the activity of HDAC and modulating the transcription of downstream genes rather than changing the microbial composition. The pro-inflammatory cytokines IL-1β and IL-18 were produced when the host was infected by the pathogens [47]. There is increasing evidence that IL-18 plays a key role in Th1-mediated immune responses [49]. Tumor necrosis factor-α (TNF-α) is a pro-inflammatory cytokine that is secreted when the host was infected by pathogens and rapidly released by mast cells after degranulation [50, 51]. It has been reported that TNF-α was produced by activation of NF-κB [52]. Previous study showed that butyrate decreased the expression of TNF-α in intestinal biopsy specimens and isolated lamina propria cells from Crohn’s disease while had no effect on the level for TNF-α in the normal person [11]. Similarly, Vinolo et al. showed that butyrate at a 1.6 mmol/l decreased the expression of *TNF-α* mRNA in lipopolysaccharide-stimulated neutrophils [53]. The inconsistent results between our study and previous studies may be explained that the piglets we used in this study kept health during the whole experimental period.

IL-10 produced by regulatory T lymphocytes, monocytes, and macrophages mainly inhibits the production of Th1 cytokines [54] and inflammatory cytokines, such as TNF-α and IFN-γ [55, 56]. Previous study showed that butyrate had the ability to facilitate extrathymic generation of regulatory T cells [57]. IFN-γ is produced by T helper-type 1 (Th1) cells, there are increasing evidences that butyrate has anti-inflammatory properties due to inhibitory on lymphocyte proliferation [58, 59], which is inconsistent with our results that butyrate decreased the gene expression of both *IFN-γ* and *IL-10*. A possible reason is that the piglets were under the low level of pathogenic stress during the suckling period. To fully understand the mechanism, in vivo pathogenic challenge model is needed in further studies.

In conclusion, this study showed that early intervention with SB significantly increased the diversity of the microbiota in the stomach and colon, and affected the gene expression of inflammatory cytokines, but had low impact on intestinal bacterial community. The results suggest that oral administration of SB may have a potentail benefit role in the health of neonatal piglets.

## Supporting Information

S1 Fig Rarefaction curves. Rarefaction curves comparing the number of sequences with the number of phylotypes found in the 16S rRNA gene libraries from microbiota in the contents in the stomach (A), ileum (B), and colon (C) of piglets in the sodium butyrate (SB) and control (CO) group. 8 and 21 represent the ages of 8 and 21 days.
(TIF)

S2 Fig Total bacteria number in the gastrointestine. The number of *16S rRNA* gene copies of total bacteria in the stomach (A), ileum (B) and colon (C) of piglets in the sodium butyrate (SB) and control (CO) groups. 8 and 21 represent the ages of 8 and 21 days respectively.
(TIF)

S1 Table List of the primers used in the present study. (DOC)

S2 Table Butyrate concentrations (μmol/g) in the stomach, ileum and colon of piglets in the sodium butyrate (SB) and control (CO) groups (n = 5). (DOC)

S3 Table Relative abundance of microbial class (percentage) in the stomach of piglets in the sodium butyrate (SB) and control (CO) groups (n = 5). (DOC)

S4 Table Relative abundance of microbial class (percentage) in the ileum of piglets in the sodium butyrate (SB) and control (CO) groups (n = 5). (DOC)

S5 Table Relative abundance of microbial class (percentage) in the colon of piglets in the sodium butyrate (SB) and control (CO) groups (n = 5). (DOC)

S6 Table Relative abundance of microbial family (percentage) in the stomach of piglets in the sodium butyrate (SB) and control (CO) groups (n = 5). (DOC)

S7 Table Relative abundance of microbial family (percentage) in the ileum of piglets in the sodium butyrate (SB) and control (CO) groups (n = 5). (DOC)

S8 Table Relative abundance of microbial family (percentage) in the colon of piglets in the sodium butyrate (SB) and control (CO) groups (n = 5). (DOC)

S9 Table Relative abundances of microbial genera (percentage) that were affected by the sodium butyrate treatment in the ileum of piglets (n = 5). (DOC)

S10 Table Relative abundances of microbial genera (percentage) that were affected by the sodium butyrate treatment in the colon of piglets. (DOC)

S11 Table Relative abundances of microbial OTUs (percentage) that were affected by the sodium butyrate treatment in the ileum of pigs (n = 5). (DOC)

S12 Table Relative abundances of microbial OTUs (percentage) that were affected by the sodium butyrate treatment in the colon of piglets (n = 5). (DOC)

## References

[1] Hendrix WF, Kelley KW, Gaskins CT, Hinrichs DJ. Porcine neonatal survival and serum gamma globulins. J Anim Sci. 1978;47(6):1281–6. 10.2134/jas1978.4761281x . 87391

[2] Rooke J, Bland I. The acquisition of passive immunity in the new-born piglet. Livest Prod Sci. 2002;78(1):13–23. 10.1016/S0301-6226(02)00182-3

[3] Wostmann BS. Germfree and gnotobiotic animal models: background and applications: CRC Press 1996;101–25.

[4] Musso G, Gambino R, Cassader M. Obesity, diabetes, and gut microbiota the hygiene hypothesis expanded? Diabetes care. 2010;33(10):2277–84. 10.2337/dc10-0556 ; PMCID: PMC2945175. 20876708 PMC2945175

[5] Hansen CHF, Nielsen DS, Kverka M, Zakostelska Z, Klimesova K, Hudcovic T, et al Patterns of early gut colonization shape future immune responses of the host. PloS One. 2012;7(3):e34043 10.1371/journal.pone.0034043 ; PMCID: PMC3313961. 22479515 PMC3313961

[6] Inman C, Haverson K, Konstantinov S, Jones P, Harris C, Smidt H, et al Rearing environment affects development of the immune system in neonates. Clin Exp Immunol. 2010;160(3):431–9. 10.1111/j.1365-2249.2010.04090.x ; PMCID: PMC2883114. 20184618 PMC2883114

[7] Kalliomaki M, Isolauri E. Pandemic of atopic diseases-a lack of microbial exposure in early infancy? Curr Drug Targets Infect Disord. 2002;2(3):193–9. 10.2174/1568005023342452 . 12462124

[8] Collado MC, Cernada M, Baüerl C, Vento M, Pérez-Martínez G. Microbial ecology and host-microbiota interactions during early life stages. Gut Microbes. 2012;3(4):352–65. 10.4161/gmic.21215 ; PMCID: PMC3463493. 22743759 PMC3463493

[9] Matamoros S, Gras-Leguen C, Le Vacon F, Potel G, de La Cochetiere M-F. Development of intestinal microbiota in infants and its impact on health. Trends Microbiol. 2013;21(4):167–73. 10.1016/j.tim.2012.12.001 . 23332725

[10] Salminen S, Bouley C, Boutron M-C, Cummings J, Franck A, Gibson G, et al Functional food science and gastrointestinal physiology and function. Br J Nutr. 1998;80(S1):S147–S71. 10.1079/BJN19980108 . 9849357

[11] Segain J, De La Blétiere DR, Bourreille A, Leray V, Gervois N, Rosales C, et al Butyrate inhibits inflammatory responses through NFκB inhibition: implications for Crohn's disease. Gut. 2000;47(3):397–403. 10.1136/gut.47.3.397 ; PMCID: PMC1728045 10940278 PMC1728045

[12] Galfi P, Bokori J. Feeding trial in pigs with a diet containing sodium n-butyrate. Acta Vet Hung. 1990;38(1–2):3–17. . 2100936

[13] Biagi G, Piva A, Moschini M, Vezzali E, Roth FX. Performance, intestinal microflora, and wall morphology of weanling pigs fed sodium butyrate. J Anim Sci. 2007;85(5):1184–91. 10.2527/jas.2006-378 . 17296766

[14] Conroy ME, Shi HN, Walker WA. The long-term health effects of neonatal microbial flora. Curr. Opin Allergy Clin Immunol. 2009;9(3):197–201. 10.1097/ACI.0b013e32832b3f1d . 19398905

[15] Saavedra JM, Dattilo AM. Early development of intestinal microbiota: implications for future health. Gastroenterol Clin N. 2012;41(4):717–31. 10.1016/j.gtc.2012.08.001 . 23101683

[16] Chinese Science and Technology Committee. Regulations for the administration of affairs concerning experimental animals. Beijing, China; 1988.

[17] Zhou L, Fang L, Sun Y, Su Y, Zhu W. Effects of the dietary protein level on the microbial composition and metabolomic profile in the hindgut of the pig. Anaerobe. 2016;38:61–9. 10.1016/j.anaerobe.2015.12.009 26723572

[18] Lane DJ, Pace B, Olsen GJ, Stahl DA, Sogin ML, Pace NR. Rapid determination of 16S ribosomal RNA sequences for phylogenetic analyses. Proc Natl Acad Sci U S A. 1985;82(20):6955–9. ; PMCID: PMC391288. 2413450 10.1073/pnas.82.20.6955 PMC391288

[19] Kroes I, Lepp PW, Relman DA. Bacterial diversity within the human subgingival crevice. Proc Natl Acad Sci U S A. 1999;96(25):14547–52. ; PMCID: PMC24473. 10588742 10.1073/pnas.96.25.14547 PMC24473

[20] Amato KR, Yeoman CJ, Kent A, Righini N, Carbonero F, Estrada A, et al Habitat degradation impacts black howler monkey (Alouatta pigra) gastrointestinal microbiomes. ISME J. 2013;7(7):1344–53. 10.1038/ismej.2013.16 ; PMCID: PMC3695285. 23486247 PMC3695285

[21] Good IJ. The population frequencies of species and the estimation of population parameters. Biometrika. 1953;40(3–4):237–64. 10.1093/biomet/40.3-4.237

[22] Schloss PD, Westcott SL, Ryabin T, Hall JR, Hartmann M, Hollister EB, et al Introducing mothur: open-source, platform-independent, community-supported software for describing and comparing microbial communities. Appl Environ Microb. 2009;75(23):7537–41. 10.1128/AEM.01541-09 ; PMCID: PMC2786419. 19801464 PMC2786419

[23] Rivas MN, Burton OT, Wise P, Zhang Y-q, Hobson SA, Lloret MG, et al A microbiota signature associated with experimental food allergy promotes allergic sensitization and anaphylaxis. J Allergy Clinl Immun. 2013;131(1):201–12. 10.1016/j.jaci.2012.10.026 ; PMCID: PMC3860814. 23201093 PMC3860814

[24] Suzuki MT, Taylor LT, DeLong EF. Quantitative analysis of small-subunit rRNA genes in mixed microbial populations via 5′-nuclease assays. Appl Environ Microb. 2000;66(11):4605–14. 10.1128/AEM.66.11.4605-4614.2000 ; PMCID: PMC92356. 11055900 PMC92356

[25] Sun Y, Zhou L, Fang L, Su Y, Zhu W. Responses in colonic microbial community and gene expression of pigs to a long-term high resistant starch diet. Front Microbiol. 2015;6:877 10.3389/fmicb.2015.00877 ; PMCID:PMC4548152. 26379652 PMC4548152

[26] Feng Z, Li T, Wu C, Tao L, Blachier F, Yin Y. Monosodium l-glutamate and dietary fat exert opposite effects on the proximal and distal intestinal health in growing pigs. Appl Physiol Nutr Me. 2014;40(4):353–63. 10.1139/apnm-2014-0434 . 25781200

[27] Tudela CV, Boudry C, Stumpff F, Aschenbach JR, Vahjen W, Zentek J, et al Down-regulation of monocarboxylate transporter 1 (MCT1) gene expression in the colon of piglets is linked to bacterial protein fermentation and pro-inflammatory cytokine-mediated signalling. Br J Nut. 2015;113(04):610–7. 10.1017/S0007114514004231 . 25656974

[28] Pié S, Lallès J, Blazy F, Laffitte J, Sève B, Oswald I. Weaning is associated with an upregulation of expression of inflammatory cytokines in the intestine of piglets. J Nutr. 2004;134(3):641–7. . 14988461 10.1093/jn/134.3.641

[29] Pieper R, Kröger S, Richter JF, Wang J, Martin L, Bindelle J, et al Fermentable fiber ameliorates fermentable protein-induced changes in microbial ecology, but not the mucosal response, in the colon of piglets. J Nutr. 2012;142(4):661–7. 10.3945/jn.111.156190 . 22357743

[30] Liu L, Liu Y, Gao F, Song G, Wen J, Guan J, et al Embryonic development and gene expression of porcine SCNT embryos treated with sodium butyrate. J Exp Biol (Mol Dev Biol). 2012;318(3):224–34. 10.1002/jez.b.22440 . 22544719

[31] Lin M, Zhang B, Yu C, Li J, Zhang L, Sun H, et al L-glutamate supplementation improves small intestinal architecture and enhances the expressions of jejunal mucosa amino acid receptors and transporters in weaning piglets. PloS One. 2014;9(11):e111950 10.1371/journal.pone.0111950 ; PMCID: PMC4219819. 25368996 PMC4219819

[32] Fang L, Jiang X, Su Y, Zhu W. Long-term intake of raw potato starch decreases back fat thickness and dressing percentage but has no effect on the longissimus muscle quality of growing–finishing pigs. Livest Sci. 2014;170:116–23. 10.1016/j.livsci.2014.10.004

[33] Li G, Yao W, Jiang H. Short-chain fatty acids enhance adipocyte differentiation in the stromal vascular fraction of porcine adipose tissue. J Nutr. 2014;144(12):1887–95. 10.3945/jn.114.198531 . 25320182

[34] Andersen CL, Jensen JL, Qrntoft TF. Normalization of real-time quantitative reverse transcription-PCR data: a model-based variance estimation approach to identify genes suited for normalization, applied to bladder and colon cancer data sets. Cancer Res. 2004;64(15):5245–5. 10.1158/0008-5472 15289330

[35] Rakoff-Nahoum S, Medzhitov R. Innate immune recognition of the indigenous microbial flora. Mucosal Immunol. 2008;1:S10–S4. 10.1038/mi.2008.49 . 19079220

[36] Rauch M, Lynch S. Probiotic manipulation of the gastrointestinal microbiota. Gut Microbes. 2010;1(5):335–8. 10.4161/gmic.1.5.13169 ; PMCID: PMC3023619. 21327043 PMC3023619

[37] Li RW, Wu S, Vi RLB, Li W, Li C. Perturbation dynamics of the rumen microbiota in response to exogenous butyrate. PloS One. 2012;7(1):e29392 10.1371/journal.pone.0029392 ; PMCID: PMC3257242. 22253719 PMC3257242

[38] Huang C, Song P, Fan P, Hou C, Thacker P, Ma X. Dietary Sodium Butyrate Decreases Postweaning Diarrhea by Modulating Intestinal Permeability and Changing the Bacterial Communities in Weaned Piglets. J Nutr. 2015;145(12):2774–80. 10.3945/jn.115.217406 . 26491121

[39] Van der Meulen R, Makras L, Verbrugghe K, Adriany T, De Vuyst L. In vitro kinetic analysis of oligofructose consumption by Bacteroides and Bifidobacterium spp. indicates different degradation mechanisms. Appl Environ Microb. 2006;72(2):1006–12. 10.1128/AEM.72.2.1006-1012.2006 ; PMCID: PMC1392924. 16461642 PMC1392924

[40] Urnbaugh PJ, Biomolecules SBd, Roscoff F. Environmental and gut bacteroidetes: the food connection. Human health and disease in a microbial world. 2011:96 10.3389/fmicb.2011.00093 ; PMCID: PMC3129010. 21747801 PMC3129010

[41] Mikkelsen LL, Naughton PJ, Hedemann MS, Jensen BB. Effects of physical properties of feed on microbial ecology and survival of Salmonella enterica serovar Typhimurium in the pig gastrointestinal tract. Appl Environ Microbiol. 2004;70(6):3485–92. 10.1128/AEM.70.6.3485-3492.2004 ; PMCID: PMC427765. 15184147 PMC427765

[42] Bugaut M. Occurrence, absorption and metabolism of short chain fatty acids in the digestive tract of mammals. Comp Biochem Physiol B. 1987;86(3):439–72. 10.1016/0305-0491(87)90433-0 . 3297476

[43] Ivarsson E, Roos S, Liu H, Lindberg J. Fermentable non-starch polysaccharides increases the abundance of Bacteroides–Prevotella–Porphyromonas in ileal microbial community of growing pigs. Animal. 2014;8(11):1777–87. 10.1017/S1751731114001827 . 25046106

[44] Sun CQ, O'Connor CJ, Turner SJ, Lewis GD, Stanley RA, Roberton AM. The effect of pH on the inhibition of bacterial growth by physiological concentrations of butyric acid: implications for neonates fed on suckled milk. Chem-Biol Interact. 1998;113(2):117–31. 10.1016/S0009-2797(98)00025-8 . 9717513

[45] Chang PV, Hao L, Offermanns S, Medzhitov R. The microbial metabolite butyrate regulates intestinal macrophage function via histone deacetylase inhibition. Proc Natl Acad Sci USA. 2014;111(6):2247–52. 10.1073/pnas.1322269111 ; PMCID: PMC3926023. 24390544 PMC3926023

[46] Zhang H, Du M, Yang Q, Zhu MJ. Butyrate suppresses murine mast cell proliferation and cytokine production through inhibiting histone deacetylase. J Nutr Biochem. 2016;27:299–306. 10.1016/j.jnutbio.2015.09.020 . 26601598

[47] Tedelind S, Westberg F, Kjerrulf M, Vidal A. Anti-inflammatory properties of the short-chain fatty acids acetate and propionate: a study with relevance to inflammatory bowel disease. World J. Gastroentero. 2007;13(20):2826–32. 10.3748/wjg.v13.i20.2826 ; PMCID: PMC4395634. 17569118 PMC4395634

[48] Hou C, Liu H, Zhang J, Zhang S, Yang F, Zeng X, et al Intestinal microbiota succession and immunomodulatory consequences after introduction of Lactobacillus reuteri I5007 in neonatal piglets. PloS one. 2015;10(3):e0119505 10.1371/journal.pone.0119505 ; PMCID: PMC4361599. 25775260 PMC4361599

[49] Micallef MJ, Ohtsuki T, Kohno K, Tanabe F, Ushio S, Namba M, et al Interferon-γ-inducing factor enhances T helper 1 cytokine production by stimulated human T cells: synergism with interleukin-12 for interferon-γ production. Eur J Immunol. 1996;26(7):1647–51. 10.1002/eji.1830260736 . 8766574

[50] Beutler B, GRAU GE. Tumor necrosis factor in the pathogenesis of infectious diseases. Crit Care Med. 1993;21(10):S423–35. . 8403980

[51] Groschwitz KR, Hogan SP. Intestinal barrier function: molecular regulation and disease pathogenesis. J Allergy Clin Immunol. 2009;124(1):3–20. 10.1016/j.jaci.2009.05.038 19560575 PMC4266989

[52] Quivy V, Van Lint C. Regulation at multiple levels of NF-kappaB-mediated transactivation by protein acetylation. Biochem pharmacol. 2004;68(6):1221–9. 10.1016/j.bcp.2004.05.039 . 15313420

[53] Vinolo MA, Rodrigues HG, Hatanaka E, Sato FT, Sampaio SC, Curi R. Suppressive effect of short-chain fatty acids on production of proinflammatory mediators by neutrophils. J Nutr Biochem. 2011;22(9):849–55. 10.1016/j.jnutbio.2010.07.009 . 21167700

[54] Mosmann TR, Sad S. The expanding universe of T-cell subsets: Th1, Th2 and more. Immunol Today. 1996;17(3):138–46. 8820272 10.1016/0167-5699(96)80606-2

[55] Ralph P, Nakoinz I, Sampson-Johannes A, Fong S, Lowe D, Min H-Y, et al IL-10, T lymphocyte inhibitor of human blood cell production of IL-1 and tumor necrosis factor. J Immunol. 1992;148(3):808–14. . 1730874

[56] Gracie JA, Forsey RJ, Chan WL, Gilmour A, Leung BP, Greer MR, et al A proinflammatory role for IL-18 in rheumatoid arthritis. J Clin Invest. 1999;104(10):1393–401. 10.1172/JCI7317 ; PMCID: PMC409841. 10562301 PMC409841

[57] Arpaia N, Campbell C, Fan X, Dikiy S, van der Veeken J, deRoos P, et al Metabolites produced by commensal bacteria promote peripheral regulatory T-cell generation. Nature. 2013;504(7480):451–5. 10.1038/nature12726 ; PMCID: PMC3869884. 24226773 PMC3869884

[58] Franklin S, Young J, Nonnecke B. Effects of ketones, acetate, butyrate, and glucose on bovine lymphocyte proliferation. J Dairy Sci. 1991;74(8):2507–14. 10.3168/jds.S0022-0302(91)78428-2 . 1918530

[59] Cavaglieri CR, Nishiyama A, Fernandes LC, Curi R, Miles EA, Calder PC. Differential effects of short-chain fatty acids on proliferation and production of pro-and anti-inflammatory cytokines by cultured lymphocytes. Life Sci. 2003;73(13):1683–90. 10.1016/S0024-3205(03)00490-9 . 12875900

