# Supplementary figures and images for: Effects of Early Intervention with Sodium Butyrate on Gut Microbiota and the Expression of Inflammatory Cytokines in Neonatal Piglets
Source: PLoS One. 2016 Sep 9;11(9):e0162461. doi: 10.1371/journal.pone.0162461 (PMC5017769; doi:10.1371/journal.pone.0162461)

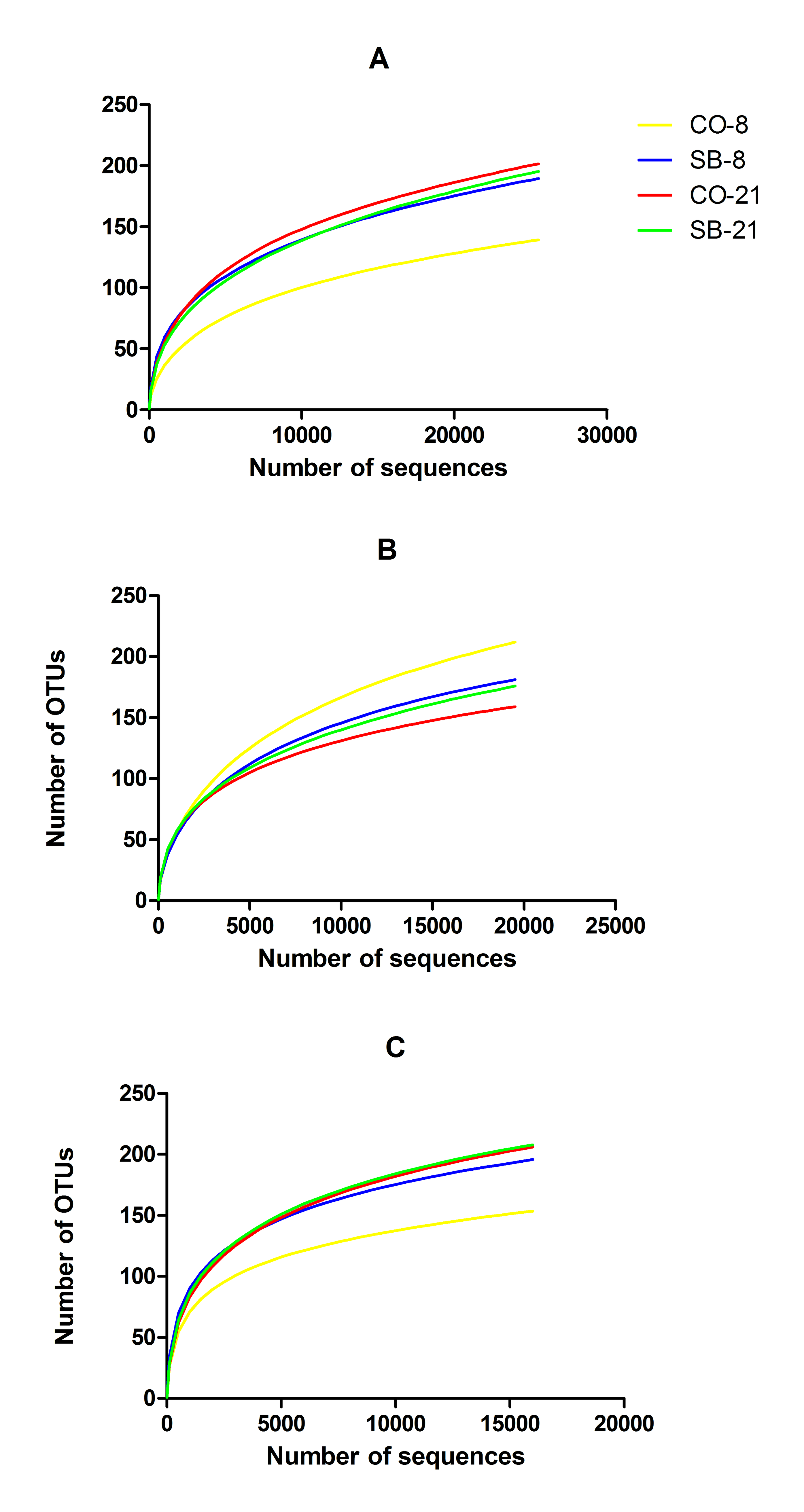

Supplement: S1 Fig — Rarefaction curves comparing the number of sequences with the number of phylotypes found in the 16S rRNA gene libraries from microbiota in the contents in the stomach (A), ileum (B), and colon (C) of piglets in the sodium butyrate (SB) and control (CO) group. 8 and 21 represent the ages of 8 and 21 days. (TIF) [file pone.0162461.s001.tif]

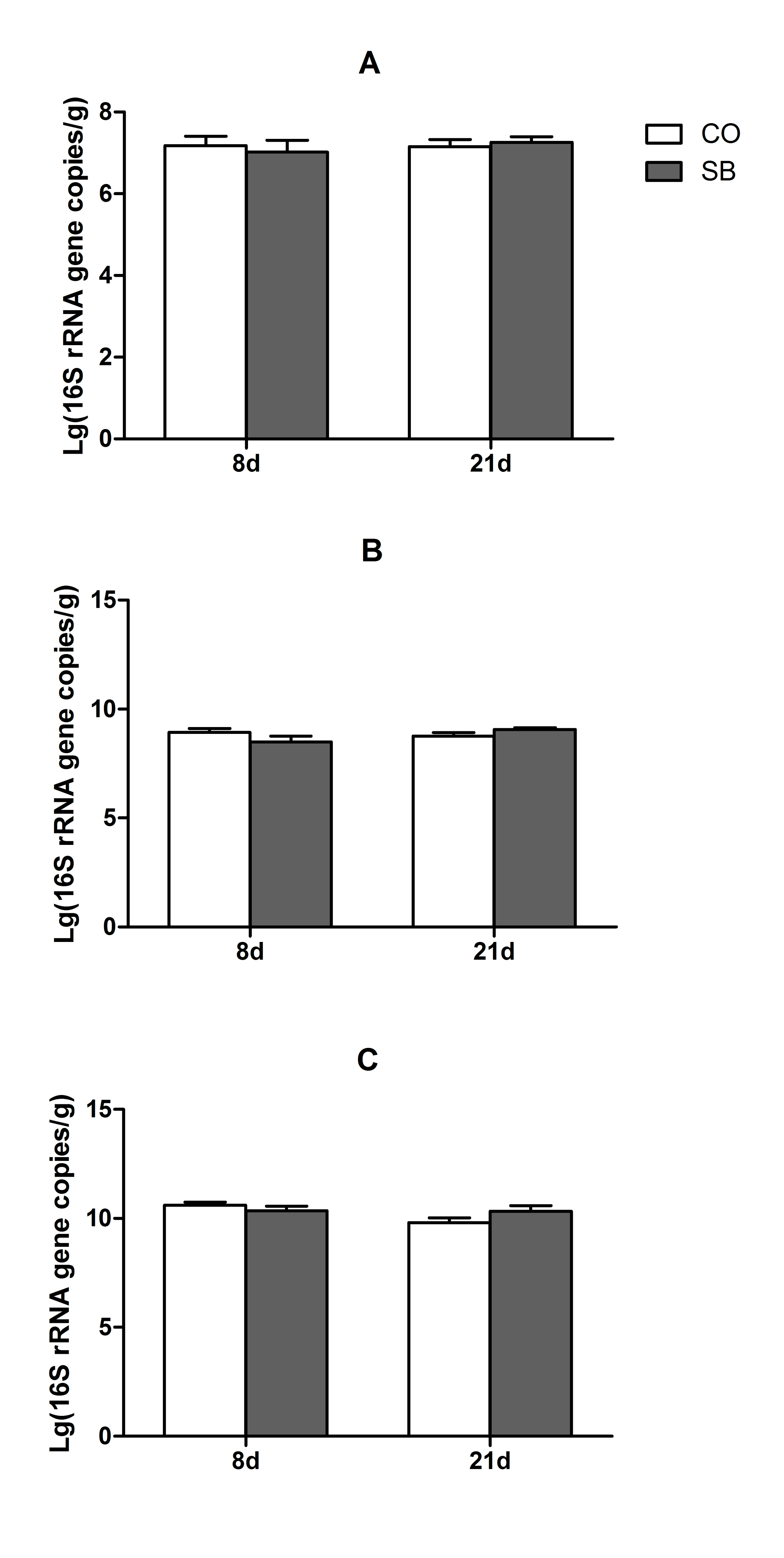

Supplement: S2 Fig — The number of 16S rRNA gene copies of total bacteria in the stomach (A), ileum (B) and colon (C) of piglets in the sodium butyrate (SB) and control (CO) groups. 8 and 21 represent the ages of 8 and 21 days respectively. (TIF) [file pone.0162461.s002.tif]
