# Supplementary material for: Effects of Early Intervention with Sodium Butyrate on Gut Microbiota and the Expression of Inflammatory Cytokines in Neonatal Piglets
Source: PLoS One. 2016 Sep 9;11(9):e0162461. doi: 10.1371/journal.pone.0162461 (PMC5017769; doi:10.1371/journal.pone.0162461)
Supplement: S1 Table — (DOC) [file pone.0162461.s003.doc]

S1 Table. List of the primers used in the present study.

| Target gene | Primer sequences | Reference |
| --- | --- | --- |
| *TNF-α* | Sense:5`-CCACGCTCTTCTGCCTACTGC-3' | [26] |
|  | Antisense:5`--GCTGTCCCTCGGCTTTGAC-3' |  |
| *IL-1β* | Sense:5`-AGTGGAGAAGCCGATGAAGA-3' | [26] |
|  | Antisense:5`-CATTGCACGTTTCAAGGATG-3' |  |
| *IFN-γ* | Sense:5`-TCCAGCGCAAAGCCATCAGTG-3’ | [27] |
|  | Antisense:5`- ATGCTCTCTGGCCTTGGAACATAGT-3’ |  |
| *IL-6* | Sense:5`-CCTCTCCGGACAAAACTGAA-3' | [26] |
|  | Antisense:5`-TCTGCCAGTACCTCCTTGCT-3' |  |
| *IL-8* | Sense:5`-TAGGACCAGAGCCAGGAAGA-3' | [26] |
|  | Antisense:5`-AGCAGGAAAACTGCCAAGAA-3' |  |
| *IL-18* | Sense:5`-TATGCCTGATTCTGACTGTT-3’ | [28] |
|  | Antisense:5`-ATGAAGACTCAAACTGTATCT-3’ |  |
| *IL-10* | Sense:5`-CTGCCTCCCACTTTCTCTTG-3' | [26] |
|  | Antisense:5`-TCAAAGGGGCTCCCTAGTTT-3' |  |
| *TGFβ* | Sense:5`-GAAGATGCTTGGAGCTGAGG-3' | [29] |
|  | Antisense:5`-TGGGACTTTGTCTTGGGAAC-3' |  |
| *HDAC1* | Sense: 5`-GTTGGAAGGGCTGATGTG-3' | [30] |
|  | Antisense: 5`-TGCTCGCTGCTGGACTTA-3' |  |
| *β-actin* | Sense: 5`-ATGCTTCTAGACGGACTGCG-3' | [31] |
|  | Antisense: 5`-GTTTCAGGAGGCTGGCATGA-3' |  |
| *GAPDH* | Sense: 5`-TTTGCGTCAGTGTCATCG-3' | [32] |
|  | Antisense: 5`-TGCTCTGCCTTGGGTAAT-3' |  |
| *18S* | Sense: 5`-CCCACGGAATCGAGAAAGAG-3' | [33] |
|  | Antisense: 5`-TTGACGGAAGGGCACCA-3' |  |
