# Supplementary material for: Effects of Early Intervention with Sodium Butyrate on Gut Microbiota and the Expression of Inflammatory Cytokines in Neonatal Piglets
Source: PLoS One. 2016 Sep 9;11(9):e0162461. doi: 10.1371/journal.pone.0162461 (PMC5017769; doi:10.1371/journal.pone.0162461)
Supplement: S2 Table — (DOC) [file pone.0162461.s004.doc]

S2 Table. Butyrate concentrations (μmol/g) in the stomach, ileum and colon of piglets in the sodium butyrate (SB) and control (CO) groups (n=5).

| Item | 8d |  | 21d |  |
| --- | --- | --- | --- | --- |
|  | CO | SB | CO | SB |
| Stomach | 0.073±0.009 | 0.095±0.033 | 0.051±0.006 | 0.157±0.060 |
| Ileum | 0.444±0.117 | 0.434±0.095 | 0.782±0.183 | 0.438±0.150 |
| Colon | 3.289±0.279 | 2.520±0.454 | 4.174±1.117 | 4.393±0.780 |
