# Supplementary material for: Effects of Early Intervention with Sodium Butyrate on Gut Microbiota and the Expression of Inflammatory Cytokines in Neonatal Piglets
Source: PLoS One. 2016 Sep 9;11(9):e0162461. doi: 10.1371/journal.pone.0162461 (PMC5017769; doi:10.1371/journal.pone.0162461)
Supplement: S3 Table — (DOC) [file pone.0162461.s005.doc]

S3 Table. Relative abundance of microbial class (percentage) in the stomach of piglets in the sodium butyrate (SB) and control (CO) groups (n=5)^1^.

| Class | | 8d |  | 21d |  | |
| --- | --- | --- | --- | --- | --- | --- |
|  |  | CO | SB | CO | | SB |
| Bacilli | 96.356±0.846 | | 88.806±2.975* | 81.544±9.214 | | 94.597±1.335 |
| Clostridia | 1.409±0.735 | | 2.032±0.142 | 11.518±8.014 | | 2.520±1.349 |
| Gammaproteobacteria | 0.639±0.116 | | 2.939±1.101 | 3.054±2.048 | | 0.926±0.213 |
| Bacteroidia | 0.496±0.092 | | 1.208±0.343 | 1.450±0.698 | | 0.540±0.083 |
| Actinobacteria | 0.274±0.107 | | 1.314±0.442* | 0.579±0.346 | | 0.524±0.222 |
| Negativicutes | 0.236±0.137 | | 1.356±0.824 | 0.357±0.140 | | 0.275±0.085 |
| Fusobacteriia | 0.223±0.090 | | 0.849±0.455 | 0.462±0.294 | | 0.127±0.018 |
| Erysipelotrichia | 0.114±0.049 | | 0.206±0.047 | 0.196±0.105 | | 0.208±0.085 |
| Flavobacteriia | 0.108±0.050 | | 0.769±0.332* | 0.439±0.273 | | 0.140±0.052 |
| norank Candidate_division_TM10 | 0.077±0.054 | | 0.148±0.099 | 0.081±0.033 | | 0.043±0.027 |
| Betaproteobacteria | 0.034±0.015 | | 0.137±0.037* | 0.196±0.105 | | 0.070±0.016 |
| norank Candidate_division_TM9 | 0.019±0.006 | | 0.147±0.086 | 0.002±0.001 | | 0.002±0.001 |

## ^1^Class with relative abundances higher than 0.05% within total bacteria were sorted and showed in the table.

## * means the significantly difference (P < 0.05) between SB group and CO group.

## ** means the significantly difference (P < 0.01) between SB group and CO group.
