# Supplementary material for: Effects of Early Intervention with Sodium Butyrate on Gut Microbiota and the Expression of Inflammatory Cytokines in Neonatal Piglets
Source: PLoS One. 2016 Sep 9;11(9):e0162461. doi: 10.1371/journal.pone.0162461 (PMC5017769; doi:10.1371/journal.pone.0162461)
Supplement: S4 Table — (DOC) [file pone.0162461.s006.doc]

**S4 Table. Relative abundance of microbial class (percentage) in the ileum of piglets in the sodium butyrate (SB) and control (CO) groups (n=5).^1^**

| Class | 8d |  | | 21d |  | |
| --- | --- | --- | --- | --- | --- | --- |
|  | CO | | SB | CO | | SB |
| Bacilli | 68.702±14.229 | | 65.657±16.119 | 82.427±5.588 | | 82.308±6.509 |
| Negativicutes | 16.398±8.569 | | 9.260± 6.360 | 4.506±1.869 | | 3.447±1.189 |
| Gammaproteobacteria | 5.596±4.612 | | 14.795± 7.766 | 1.052±0.379 | | 0.760±0.487 |
| Clostridia | 3.459±1.303 | | 5.090± 2.435 | 6.006±1.965 | | 9.054±3.744 |
| Fusobacteriia | 3.329±2.454 | | 2.297± 1.433 | 0.611±0.319 | | 0.130±0.026 |
| Actinobacteria | 0.908±0.226 | | 1.093± 0.439 | 3.941±2.210 | | 2.880±0.791 |
| Bacteroidia | 0.769±0.214 | | 0.451± 0.249 | 0.055±0.019 | | 0.070±0.022 |
| norank Candidate_division_TM7 | 0.396±0.186 | | 0.402± 0.250 | 0.794±0.457 | | 0.611±0.453 |
| Erysipelotrichia | 0.096±0.037 | | 0.167± 0.047 | 0.459±0.300 | | 0.632±0.473 |
| Betaproteobacteria | 0.071±0.020 | | 0.173± 0.050 | 0.036±0.011 | | 0.038±0.019 |
| Alphaproteobacteria | 0.064±0.017 | | 0.208± 0.090 | 0.035±0.016 | | 0.046±0.026 |
| Mollicutes | 0.026±0.024 | | 0.050± 0.030 | 0.009±0.005 | | 0.003±0.002 |
| Acidimicrobiia | 0.018±0.013 | | 0.053± 0.033 | 0.000±0.000 | | 0.000±0.000 |
| Flavobacteriia | 0.009±0.004 | | 0.170± 0.076** | 0.007±0.004 | | 0.000±0.000 |

## ^1^Class with relative abundances higher than 0.05% within total bacteria were sorted and showed in the table.

## * means the significantly difference (P < 0.05) between SB group and CO group.

## ** means the significantly difference (P < 0.01) between SB group and CO group.
