# Supplementary material for: Effects of Early Intervention with Sodium Butyrate on Gut Microbiota and the Expression of Inflammatory Cytokines in Neonatal Piglets
Source: PLoS One. 2016 Sep 9;11(9):e0162461. doi: 10.1371/journal.pone.0162461 (PMC5017769; doi:10.1371/journal.pone.0162461)
Supplement: S5 Table — (DOC) [file pone.0162461.s007.doc]

S5 Table. Relative abundance of microbial class (percentage) in the colon of piglets in the sodium butyrate (SB) and control (CO) groups (n=5).

| Class | 8d |  | | 21d |  | |
| --- | --- | --- | --- | --- | --- | --- |
|  | CO | | SB | CO | | SB |
| Bacteroidia | 64.960±3.542 | | 41.254±4.470** | 19.040± 6.005 | | 29.486±7.001 |
| Clostridia | 19.702±3.439 | | 25.377±1.307 | 49.980±15.148 | | 45.011±11.916 |
| Bacilli | 7.371±1.928 | | 12.975±4.218 | 18.160± 9.403 | | 17.447± 6.248 |
| Negativicutes | 2.631±0.983 | | 7.718±1.216* | 5.649± 2.656 | | 2.526± 1.327 |
| Fusobacteriia | 2.274±1.230 | | 7.628±5.487 | 0.220± 0.131 | | 0.466± 0.282 |
| Spirochaetes | 0.987±0.981 | | 0.018±0.017 | 0.036± 0.033 | | 0.004± 0.002 |
| Gammaproteobacteria | 0.908±0.609 | | 2.776±1.668 | 0.687± 0.491 | | 1.020± 0.885 |
| Verrucomicrobiae | 0.351±0.254 | | 0.852±0.800 | 0.000± 0.000 | | 0.000± 0.000 |
| Erysipelotrichia | 0.306±0.230 | | 0.817±0.463 | 1.298± 0.592 | | 2.759± 0.753 |
| Cyanobacteria | 0.144±0.144 | | 0.000±0.000 | 0.078± 0.075 | | 0.020± 0.014 |
| Deltaproteobacteria | 0. 960±0.028 | | 0.211±0.060 | 0.195± 0.082 | | 0.190± 0.103 |
| Actinobacteria | 0.079±0.018 | | 0.287±0.082* | 2.377± 1.440 | | 0.631± 0.183 |
| Synergistia | 0.024±0.015 | | 0.001±0.001 | 2.071± 1.832 | | 0.298± 0.242 |
| Mollicutes | 0.001±0.001 | | 0.023±0.023 | 0.154± 0.102 | | 0.098± 0.096 |

## ^1^Class with relative abundances higher than 0.05% within total bacteria were sorted and showed in the table.

## * means the significantly difference (P < 0.05) between SB group and CO group.

## ** means the significantly difference (P < 0.01) between SB group and CO group.

## 
