# Supplementary material for: Effects of Early Intervention with Sodium Butyrate on Gut Microbiota and the Expression of Inflammatory Cytokines in Neonatal Piglets
Source: PLoS One. 2016 Sep 9;11(9):e0162461. doi: 10.1371/journal.pone.0162461 (PMC5017769; doi:10.1371/journal.pone.0162461)
Supplement: S6 Table — (DOC) [file pone.0162461.s008.doc]

S6 Table. Relative abundance of microbial family (percentage) in the stomach of piglets in the sodium butyrate (SB) and control (CO) groups (n=5)^1^.

| Family | 8d |  | | | 21d |  | |
| --- | --- | --- | --- | --- | --- | --- | --- |
|  | CO | | SB | CO | | | SB |
| Lactobacillaceae | 95.149±0.922 | | 83.764±5.205* | 79.412±9.890 | | | 92.838±1.200 |
| Streptococcaceae | 0.956±0.309 | | 4.144±2.228 | 1.084±0.414 | | | 1.162±0.253 |
| Peptostreptococcaceae | 0.612±0.396 | | 0.963±0.238 | 1.797±1.416 | | | 1.488±0.850 |
| Clostridiaceae_1 | 0.608±0.322 | | 0.646±0.121 | 8.834±8.228 | | | 0.718±0.430 |
| Moraxellaceae | 0.307±0.072 | | 1.436±0.576 | 0.896±0.597 | | | 0.255±0.071 |
| Pasteurellaceae | 0.276±0.061 | | 1.438±0.544* | 2.122±1.445 | | | 0.646±0.172 |
| Veillonellaceae | 0.220±0.131 | | 1.340±0.825 | 0.336±0.132 | | | 0.261±0.084 |
| Porphyromonadaceae | 0.206±0.085 | | 0.736±0.248 | 0.473±0.309 | | | 0.125±0.007 |
| unclassified Lactobacillales | 0.137±0.045 | | 0.240±0.137 | 0.134±0.049 | | | 0.235±0.097 |
| Bacteroidaceae | 0.132±0.034 | | 0.053±0.027 | 0.179±0.094 | | | 0.088±0.047 |
| Fusobacteriaceae | 0.126±0.060 | | 0.227±0.071 | 0.174±0.090 | | | 0.068±0.019 |
| Prevotellaceae | 0.123±0.017 | | 0.361±0.098 | 0.644±0.237 | | | 0.278±0.089 |
| Flavobacteriaceae | 0.108±0.050 | | 0.769±0.332* | 0.439±0.273 | | | 0.140±0.052 |
| Erysipelotrichaceae | 0.114±0.049 | | 0.206±0.047 | 0.196±0.105 | | | 0.208±0.085 |
| Leptotrichiaceae | 0.096±0.033 | | 0.621±0.445 | 0.288±0.207 | | | 0.060±0.033 |
| Micrococcaceae | 0.081±0.028 | | 0.652±0.251** | 0.154±0.044 | | | 0.294±0.131 |
| norank Candidate_division_TM7 | 0.077±0.054 | | 0.148±0.099 | 0.086±0.032 | | | 0.043±0.027 |
| Corynebacteriaceae | 0.078±0.039 | | 0.426±0.200 | 0.206±0.163 | | | 0.139±0.078 |
| Ruminococcaceae | 0.073±0.014 | | 0.254±0.095 | 0.387±0.147 | | | 0.082±0.025 |
| Actinomycetaceae | 0.073±0.048 | | 0.091±0.041 | 0.170±0.126 | | | 0.039±0.009 |
| Lachnospiraceae | 0.072±0.017 | | 0.109±0.026 | 0.281±0.148 | | | 0.120±0.034 |
| Family_XI | 0.054±0.021 | | 0.116±0.030 | 0.174±0.140 | | | 0.083±0.036 |
| Aerococcaceae | 0.039±0.011 | | 0.304±0.137** | 0.363±0.318 | | | 0.199±0.115 |
| S24-7 | 0.025±0.013 | | 0.028±0.010 | 0.114±0.057 | | | 0.031±0.016 |
| Neisseriaceae | 0.022±0.011 | | 0.063±0.015 | 0.172±0.120 | | | 0.053±0.017 |
| Staphylococcaceae | 0.024±0.013 | | 0.069±0.011 | 0.349±0.332 | | | 0.053±0.017 |
| norank Candidate_division_TM8 | 0.019±0.006 | | 0.147±0.086 | 0.002±0.001 | | | 0.002±0.001 |
| Enterococcaceae | 0.019±0.015 | | 0.065±0.045 | 0.024±0.008 | | | 0.008±0.004 |
| Carnobacteriaceae | 0.008±0.003 | | 0.044±0.021 | 0.119±0.115 | | | 0.026±0.010 |
| Leuconostocaceae | 0.003±0.001 | | 0.094±0.044 | 0.044±0.028 | | | 0.029±0.020 |

## ^1^Family with relative abundances higher than 0.05% within total bacteria were sorted and showed in the table.

## * means the significantly difference (P < 0.05) between SB group and CO group.

## ** means the significantly difference (P < 0.01) between SB group and CO group.
