# Supplementary material for: Effects of Early Intervention with Sodium Butyrate on Gut Microbiota and the Expression of Inflammatory Cytokines in Neonatal Piglets
Source: PLoS One. 2016 Sep 9;11(9):e0162461. doi: 10.1371/journal.pone.0162461 (PMC5017769; doi:10.1371/journal.pone.0162461)
Supplement: S7 Table — (DOC) [file pone.0162461.s009.doc]

S7 Table. Relative abundance of microbial family (percentage) in the ileum of piglets in the sodium butyrate (SB) and control (CO) groups (n=5).

| Family | 8d |  | | | 21d |  | |
| --- | --- | --- | --- | --- | --- | --- | --- |
|  | CO | | SB | CO | | | SB |
| Lactobacillaceae | 62.455±14.538 | | 56.076±18.134 | 68.619±13.038 | | | 64.317±6.911 |
| Veillonellaceae | 16.300± 8.585 | | 9.234± 6.352 | 4.499± 1.868 | | | 3.440±1.186 |
| Streptococcaceae | 5.494± 1.446 | | 8.240± 4.168 | 10.924± 5.485 | | | 16.346±3.092 |
| Pasteurellaceae | 4.839± 4.556 | | 11.974± 7.233 | 0.555± 0.365 | | | 0.488±0.461 |
| Fusobacteriaceae | 3.312± 2.450 | | 2.230± 1.433 | 0.604± 0.321 | | | 0.118±0.019 |
| Peptostreptococcaceae | 1.552± 0.890 | | 3.527± 2.244 | 1.200± 0.484 | | | 7.288±3.372 |
| Clostridiaceae_1 | 0.688± 0.372 | | 0.833± 0.628 | 2.051± 1.897 | | | 0.549±0.284 |
| Family_XI | 0.537± 0.183 | | 0.758± 0.474 | 1.425± 1.080 | | | 0.610±0.132 |
| Ruminococcaceae | 0.535± 0.238 | | 0.217± 0.082 | 0.521± 0.351 | | | 0.217±0.053 |
| Lachnospiraceae | 0.439± 0.165 | | 0.368± 0.243 | 1.349± 0.605 | | | 0.661±0.096 |
| norank Candidate_division_TM7 | 0.396± 0.186 | | 0.402± 0.250 | 0.794± 0.457 | | | 0.611±0.453 |
| Corynebacteriaceae | 0.368± 0.125 | | 0.602± 0.357 | 1.084± 0.571 | | | 1.098±0.301 |
| Actinomycetaceae | 0.324± 0.135 | | 0.156± 0.045 | 2.320± 1.489 | | | 1.351±0.928 |
| Bacteroidaceae | 0.322± 0.084 | | 0.210± 0.128 | 0.020± 0.009 | | | 0.013±0.003 |
| Moraxellaceae | 0.295± 0.161 | | 0.845± 0.332 | 0.055± 0.028 | | | 0.018±0.016 |
| Prevotellaceae | 0.230± 0.059 | | 0.138± 0.053 | 0.024± 0.012 | | | 0.021±0.008 |
| Enterobacteriaceae | 0.220± 0.103 | | 0.488± 0.252 | 0.117±0.076 | | | 0.085±0.029 |
| Pseudomonadaceae | 0.200± 0.045 | | 1.457± 0.791 | 0.324±0.172 | | | 0.166±0.068 |
| Aerococcaceae | 0.120± 0.032 | | 0.309± 0.193 | 0.801±0.721 | | | 0.248±0.065 |
| unclassified Lactobacillales | 0.097± 0.035 | | 0.116± 0.093 | 0.532±0.379 | | | 0.688±0.167 |
| Micrococcaceae | 0.098± 0.031 | | 0.197± 0.113 | 0.183±0.081 | | | 0.192±0.045 |
| Erysipelotrichaceae | 0.096± 0.037 | | 0.167± 0.047 | 0.459±0.300 | | | 0.632±0.473 |
| Acidaminococcaceae | 0.097± 0.051 | | 0.026± 0.014 | 0.006±0.001 | | | 0.008±0.003 |
| Family_XIII | 0.077± 0.045 | | 0.042± 0.018 | 0.186±0.115 | | | 0.172±0.053 |
| S24-7 | 0.072± 0.055 | | 0.017± 0.013 | 0.004±0.001 | | | 0.013±0.009 |
| Staphylococcaceae | 0.049± 0.037 | | 0.045± 0.011 | 0.332±0.323 | | | 0.066±0.023 |
| Alcaligenaceae | 0.040± 0.010 | | 0.079± 0.028 | 0.026±0.011 | | | 0.029±0.022 |
| Microbacteriaceae | 0.030± 0.024 | | 0.030± 0.014 | 0.093±0.053 | | | 0.080±0.063 |
| Enterococcaceae | 0.033± 0.017 | | 0.097± 0.057 | 0.332±0.213 | | | 0.099±0.049 |
| Coriobacteriaceae | 0.023± 0.020 | | 0.005± 0.003 | 0.135±0.082 | | | 0.085±0.033 |
| Carnobacteriaceae | 0.023± 0.011 | | 0.065± 0.047 | 0.092±0.085 | | | 0.033±0.018 |
| Mycobacteriaceae | 0.021± 0.011 | | 0.056± 0.035 | 0.003±0.002 | | | 0.003±0.002 |
| Leptotrichiaceae | 0.016± 0.007 | | 0.067± 0.050 | 0.007±0.004 | | | 0.012±0.012 |
| Flavobacteriaceae | 0.009± 0.004 | | 0.170± 0.076** | 0.007±0.004 | | | 0.000±0.000 |
| Dermabacteraceae | 0.000± 0.000 | | 0.010± 0.009 | 0.069±0.045 | | | 0.005±0.003 |

^1^Family with relative abundances higher than 0.05% within total bacteria were sorted and showed in the table.

* means the significantly difference (P < 0.05) between SB group and CO group.

** means the significantly difference (P < 0.01) between SB group and CO group.
