# Supplementary material for: Effects of Early Intervention with Sodium Butyrate on Gut Microbiota and the Expression of Inflammatory Cytokines in Neonatal Piglets
Source: PLoS One. 2016 Sep 9;11(9):e0162461. doi: 10.1371/journal.pone.0162461 (PMC5017769; doi:10.1371/journal.pone.0162461)
Supplement: S8 Table — (DOC) [file pone.0162461.s010.doc]

S8 Table. Relative abundance of microbial family (percentage) in the colon of piglets in the sodium butyrate (SB) and control (CO) groups (n=5).

| Family | 8d |  | | 21d |  | |
| --- | --- | --- | --- | --- | --- | --- |
|  | CO | | SB | CO | | SB |
| Bacteroidaceae | 32.177±9.916 | | 16.772±5.320 | 1.995± 1.533 | | 13.911± 7.567 |
| Ruminococcaceae | 13.726±2.766 | | 14.292±2.064 | 42.990±15.503 | | 37.966±13.203 |
| Prevotellaceae | 13.118±7.975 | | 11.723±4.119 | 6.113±4.856 | | 5.590± 3.103 |
| Rikenellaceae | 12.099±7.863 | | 3.536±1.791 | 0.597±0.307 | | 1.037± 0.615 |
| Lactobacillaceae | 6.609±1.669 | | 10.998±4.417 | 17.527±9.343 | | 16.968± 6.025 |
| Porphyromonadaceae | 5.277±1.740 | | 1.192±0.355 | 0.577±0.494 | | 0.575± 0.303 |
| Lachnospiraceae | 3.159±1.035 | | 6.701±1.136 | 4.403±1.363 | | 4.442± 1.322 |
| Fusobacteriaceae | 2.273±1.230 | | 7.628±5.487 | 0.220±0.131 | | 0.466± 0.282 |
| S24-7 | 2.190±1.607 | | 7.105±3.751 | 9.708±4.643 | | 8.241± 4.637 |
| Acidaminococcaceae | 2.107±1.038 | | 5.691±1.656 | 3.073±1.648 | | 2.353± 1.202 |
| Clostridiaceae_1 | 1.597±0.923 | | 1.741±0.519 | 0.060±0.041 | | 0.303± 0.143 |
| Spirochaetaceae | 0.987±0.981 | | 0.018±0.017 | 0.036±0.033 | | 0.004± 0.002 |
| Pasteurellaceae | 0.748±0.510 | | 2.179±1.463 | 0.019±0.006 | | 0.035± 0.026 |
| Streptococcaceae | 0.684±0.394 | | 1.841±0.813 | 0.576±0.218 | | 0.434± 0.238 |
| Veillonellaceae | 0.523±0.118 | | 2.028±0.622 | 2.576±2.385 | | 0.173± 0.129 |
| Family_XIII | 0.397±0.208 | | 1.311±0.916 | 1.080±0.219 | | 0.967± 0.435 |
| Verrucomicrobiaceae | 0.351±0.254 | | 0.852±0.800 | 0.000±0.000 | | 0.000± 0.000 |
| Erysipelotrichaceae | 0.306±0.230 | | 0.817±0.463 | 1.298±0.592 | | 2.759± 0.753 |
| Defluviitaleaceae | 0.259±0.092 | | 0.387±0.092 | 0.257±0.070 | | 0.197± 0.125 |
| vadinBB60 | 0.253±0.235 | | 0.062±0.042 | 0.028±0.025 | | 0.014± 0.009 |
| Peptostreptococcaceae | 0.235±0.147 | | 0.794±0.294 | 0.412±0.194 | | 0.689± 0.098 |
| norank Gastranaerophilales | 0.144±0.144 | | 0.000±0.000 | 0.078±0.075 | | 0.020± 0.014 |
| Enterobacteriaceae | 0.141±0.090 | | 0.582±0.329 | 0.625±0.505 | | 0.984± 0.885 |
| Desulfovibrionaceae | 0.096±0.028 | | 0.185±0.071 | 0.195±0.082 | | 0.176± 0.108 |
| unclassified Bacteroidetes | 0.071±0.069 | | 0.032±0.028 | 0.007±0.004 | | 0.021± 0.013 |
| unclassified Lactobacillales | 0.059±0.042 | | 0.073±0.023 | 0.011±0.004 | | 0.039± 0.026 |
| Coriobacteriaceae | 0.046±0.014 | | 0.124±0.067 | 2.083±1.472 | | 0.470± 0.166 |
| Eubacteriaceae | 0.037±0.037 | | 0.001±0.001 | 0.082±0.055 | | 0.006± 0.004 |
| Synergistaceae | 0.024±0.018 | | 0.001±0.001 | 2.071±1.832 | | 0.298± 0.242 |
| Actinomycetaceae | 0.016±0.006 | | 0.078±0.034 | 0.088±0.037 | | 0.077± 0.060 |
| Corynebacteriaceae | 0.009±0.005 | | 0.066±0.032 | 0.164±0.080 | | 0.072± 0.048 |
| Christensenellaceae | 0.009±0.009 | | 0.025±0.010 | 0.542±0.410 | | 0.365± 0.162 |
| Peptococcaceae | 0.008±0.005 | | 0.023±0.023 | 0.096±0.041 | | 0.046± 0.027 |

## ^1^Family with relative abundances higher than 0.05% within total bacteria were sorted and showed in the table.

## * means the significantly difference (P < 0.05) between SB group and CO group.

## ** means the significantly difference (P < 0.01) between SB group and CO group.
