# Supplementary material for: Effects of Early Intervention with Sodium Butyrate on Gut Microbiota and the Expression of Inflammatory Cytokines in Neonatal Piglets
Source: PLoS One. 2016 Sep 9;11(9):e0162461. doi: 10.1371/journal.pone.0162461 (PMC5017769; doi:10.1371/journal.pone.0162461)
Supplement: S9 Table — (DOC) [file pone.0162461.s011.doc]

S9 Table. Relative abundances of microbial genera (percentage) that were affected by the sodium butyrate treatment in the ileum of piglets (n=5).

| **Genus** | **8d** | |  | | **21d** | |  |
| --- | --- | --- | --- | --- | --- | --- | --- |
|  | **CO** | **SB** | | **CO** | | **SB** | |
| *Acetitomaculum* | 0.009±0.009 | 0.010±0.010 | | 0.051±0.043 | | 0.015±0.008 | |
| *Acinetobacter* | 0.017±0.009 | 0.051±0.020 | | 0.017±0.008 | | 0.008±0.003 | |
| *Actinobacillus* | 3.241±3.022 | 4.401±4.012 | | 0.468±0.318 | | 0.362±0.347 | |
| *Actinomyces* | 0.117±0.054 | 0.070±0.033 | | 2.138±1.386 | | 1.065±0.650 | |
| *Alloprevotella* | 0.050±0.012 | 0.030±0.008 | | 0.009±0.006 | | 0.003±0.003 | |
| *Anaerococcus* | 0.003±0.002 | 0.015±0.010 | | 0.262±0.261 | | 0.010±0.008 | |
| *Arcanobacterium* | 0.201±0.111 | 0.081±0.015 | | 0.169±0.095 | | 0.186±0.093 | |
| *Bacteroides* | 0.322±0.084 | 0.210±0.128 | | 0.020±0.009 | | 0.010±0.004 | |
| *Bergeyella* | 0.006±0.003 | 0.156±0.073 | | 0.005±0.003 | | 0.000±0.000 | |
| *Blautia* | 0.014±0.009 | 0.004±0.003 | | 0.053±0.048 | | 0.015±0.002 | |
| *Clostridium_sensu_stricto_*1 | 0.385±0.116 | 0.497±0.356 | | 0.112±0.054 | | 0.450±0.241 | |
| *Corynebacterium* | 0.368±0.125 | 0.602±0.357 | | 1.084±0.571 | | 1.038±0.241 | |
| *Dermabacter* | 0.000±0.000 | 0.001±0.001 | | 0.056±0.046 | | 0.011±0.010 | |
| *Enhydrobacter* | 0.001±0.001 | 0.055±0.047 | | 0.006±0.005 | | 0.006±0.006 | |
| *Escherichia-Shigella* | 0.178±0.099 | 0.428±0.233 | | 0.100±0.066 | | 0.048±0.007 | |
| *Facklamia* | 0.022±0.011 | 0.029±0.016 | | 0.122±0.111 | | 0.045±0.017 | |
| *Faecalibacterium* | 0.085±0.019 | 0.059±0.033 | | 0.003±0.002 | | 0.604±0.321 | |
| *Fusobacterium* | 3.312±2.450 | 2.230±1.433 | | 0.604±0.321 | | 0.237±0.119 | |
| *Gemella* | 0.425±1.346 | 0.669±0.453 | | 0.778±0.522 | | 0.631±0.174 | |
| *Globicatella* | 0.079±0.018 | 0.257±0.175 | | 0.399±0.334 | | 0.203±0.052 | |
| *Granulicatella* | 0.019±0.011 | 0.051±0.043 | | 0.066±0.061 | | 0.040±0.018 | |
| *Haemophilus* | 0.109±0.079 | 1.443±0.864 | | 0.056±0.042 | | 0.004±0.002 | |
| *Helcococcus* | 0.071±0.038 | 0.046±0.029 | | 0.142±0.108 | | 0.077±0.026 | |
| *Howardella* | 0.051±0.027 | 0.011±0.008 | | 0.057±0.021 | | 0.106±0.048 | |
| *Ignavigranum* | 0.012±0.010 | 0.008±0.005 | | 0.171±0.171 | | 0.006±0.002 | |
| *Jeotgalicoccus* | 0.005±0.005 | 0.005±0.002 | | 0.076±0.072 | | 0.008±0.005 | |
| *Johnsonella* | 0.000±0.000 | 0.002±0.002 | | 0.065±0.038 | | 0.087±0.076 | |
| *Lactobacillus* | 62.45±14.54 | 56.07±18.13 | | 68.62±13.04 | | 54.69±11.01 | |
| *Lactococcus* | 0.012±0.011 | 0.065±0.027 | | 0.014±0.013 | | 0.001±0.001 | |
| *Leptotrichia* | 0.014±0.007 | 0.059±0.050 | | 0.006±0.004 | | 0.010±0.009 | |
| *Leucobacter* | 0.012±0.006 | 0.002±0.002 | | 0.076±0.046 | | 0.053±0.027 | |
| *Megamonas* | 0.146±0.047 | 0.083±0.055 | | 0.019±0.018 | | 0.000±0.000 | |
| *Moraxella* | 0.275±0.153 | 0.730±0.283 | | 0.031±0.020 | | 0.010±0.007 | |
| *Mycobacterium* | 0.021±0.011 | 0.056±0.035 | | 0.003±0.002 | | 0.005±0.003 | |
| norank Bacteroidales S24-7 | 0.072±0.055 | 0.017±0.011 | | 0.004±0.001 | | 0.014±0.007 | |
| norank Candidate_division_TM7 | 0.396±0.186 | 0.402±0.250 | | 0.794±0.457 | | 0.554±0.356 | |
| *Nosocomiicoccus* | 0.029±0.029 | 0.011±0.009 | | 0.235±0.235 | | 0.011±0.010 | |
| *Parvimonas* | 0.002±0.002 | 0.001±0.001 | | 0.051±0.027 | | 0.038±0.015 | |
| *Pasteurella* | 1.428±1.396 | 6.107±4.583 | | 0.023±0.020 | | 0.012±0.008 | |
| *Peptoniphilus* | 0.025±0.015 | 0.007±0.007 | | 0.103±0.103 | | 0.011±0.008 | |
| *Peptostreptococcus* | 0.421±0.176 | 0.263±0.159 | | 0.765±0.330 | | 0.470±0.105 | |
| *Phascolarctobacterium* | 0.097±0.051 | 0.026±0.014 | | 0.005±0.002 | | 0.005±0.003 | |
| *Pseudomonas* | 0.200±0.045 | 1.457±0.791 | | 0.324±0.172 | | 0.167±0.052 | |
| *Rothia* | 0.072±0.026 | 0.163±0.104 | | 0.170±0.075 | | 0.183±0.036 | |
| *Sarcina* | 0.297±0.280 | 0.323±0.280 | | 1.938±1.900 | | 0.000±0.000* | |
| *Solobacterium* | 0.004±0.003 | 0.000±0.000 | | 0.093±0.071 | | 0.007±0.004 | |
| *Streptococcus* | 5.482±1.436 | 8.175±4.163 | | 10.91±5.481 | | 27.73±11.63 | |
| *Turicibacter* | 0.049±0.023 | 0.088±0.027 | | 0.072±0.025 | | 0.399±0.312 | |
| uncultured Clostridiales Family _ XI | 0.009±0.006 | 0.020±0.019 | | 0.080±0.080 | | 0.002±0.002 | |
| uncultured Clostridiales *Family _XIII* | 0.023±0.022 | 0.025±0.016 | | 0.060±0.047 | | 0.107±0.043 | |
| uncultured Erysipelotrichaceae | 0.030±0.011 | 0.078±0.037 | | 0.241±0.166 | | 0.153±0.078 | |
| uncultured Prevotellaceae | 0.151±0.038 | 0.092±0.043 | | 0.003±0.002 | | 0.002±0.001 | |
| uncultured Ruminococcaceae | 0.240±0.176 | 0.077±0.017 | | 0.273±0.129 | | 0.145±0.030 | |
| *Veillonella* | 16.15±8.560 | 9.144±6.341 | | 4.479±1.867 | | 2.889±1.070 | |
| unclassified Aerococcaceae | 0.007±0.003 | 0.002±0.002 | | 0.097±0.094 | | 0.009±0.005 | |
| unclassified Alcaligenaceae | 0.026±0.007 | 0.074±0.029 | | 0.026±0.011 | | 0.041±0.020 | |
| unclassified Bacteroidales | 0.071±0.031 | 0.043±0.033 | | 0.001±0.001 | | 0.000±0.000 | |
| unclassified Coriobacteriaceae | 0.014±0.011 | 0.003±0.002 | | 0.051±0.036 | | 0.022±0.002 | |
| unclassified Enterococcaceae | 0.026±0.023 | 0.065±0.057 | | 0.326±0.214 | | 0.071±0.042 | |
| unclassified Lachnospiraceae | 0.290±0.130 | 0.287±0.202 | | 1.013±0.445 | | 0.448±0.023 | |
| unclassified Lactobacillales | 0.097±0.031 | 0.116±0.093 | | 0.532±0.379 | | 0.643±0.137 | |
| unclassified Pasteurellaceae | 0.061±0.060 | 0.023±0.023 | | 0.007±0.004 | | 0.014±0.013 | |
| unclassified Ruminococcaceae | 0.067±0.022 | 0.026±0.017 | | 0.007±0.006 | | 0.007±0.003 | |

## ^1^Genera with relative abundances higher than 0.05% within total bacteria were sorted and showed in the table.

## * means the significantly difference (*P* < 0.05) between SB group and CO group.

## ** means the significantly difference (*P* < 0.01) between SB group and CO group.
