# Supplementary material for: Effects of Early Intervention with Sodium Butyrate on Gut Microbiota and the Expression of Inflammatory Cytokines in Neonatal Piglets
Source: PLoS One. 2016 Sep 9;11(9):e0162461. doi: 10.1371/journal.pone.0162461 (PMC5017769; doi:10.1371/journal.pone.0162461)
Supplement: S10 Table — (DOC) [file pone.0162461.s012.doc]

S10 Table. Relative abundances of microbial genera (percentage) that were affected by the sodium butyrate treatment in the colon of piglets.

| **Genus** | **8d** |  | | | **21d** | |  |  |
| --- | --- | --- | --- | --- | --- | --- | --- | --- |
|  | **CO** | | **SB** | **CO** | | **SB** | | |
| *Actinobacillus* | 0.305±0.210 | | 0.282±0.083 | 0.014±0.005 | | 0.030±0.015 | | |
| *Actinomyces* | 0.012±0.004 | | 0.068±0.032 | 0.085±0.034 | | 0.078±0.046 | | |
| *Akkermansia* | 0.351±0.254 | | 0.851±0.800 | 0.000±0.000 | | 0.000±0.000 | | |
| *Alistipes* | 3.891±1.630 | | 2.786±1.109 | 0.151±0.069 | | 0.260±0.168 | | |
| *Alloprevotella* | 1.387±1.364 | | 3.768±2.127 | 1.271±0.922 | | 0.999±0.706 | | |
| *Anaerotruncus* | 4.701±2.454 | | 4.239±1.678 | 2.285±0.986 | | 12.71±6.914 | | |
| *Anaerovibrio* | 0.000±0.000 | | 0.351±0.348 | 2.306±2.295 | | 0.008±0.007 | | |
| *Bacteroides* | 32.18±9,916 | | 16.77±5.320 | 1.995±1.533 | | 11.56±6.315 | | |
| *Blautia* | 0.430±0.204 | | 0.622±0.238 | 0.759±0.240 | | 1.420±0.656 | | |
| *Butyricimonas* | 3.579±1.803 | | 0.436±0.131 | 0.005±0.002 | | 0.058±0.033 | | |
| *Cloacibacillus* | 0.234±0.018 | | 0.001±0.001 | 1.095±0.868 | | 0.018±0.007 | | |
| *Clostridium_sensu_stricto_*1 | 1.480±0.882 | | 1.654±0.506 | 0.059±0.041 | | 0.244±0.126 | | |
| *Collinsella* | 0.008±0.005 | | 0.081±0.074 | 2.041±1.478 | | 0.362±0.153 | | |
| *Coprococcus* | 0.052±0.026 | | 0.109±0.035 | 0.115±0.048 | | 0.074±0.030 | | |
| *Corynebacterium* | 0.010±0.006 | | 0.066±0.032 | 0.164±0.080 | | 0.098±0.046 | | |
| *Desulfovibrio* | 0.076±0.023 | | 0.163±0.078 | 0.165±0.086 | | 0.118±0.069 | | |
| dgA-11_gut_group | 7.097±7/096 | | 0.087±0.084 | 0.022±0.009 | | 0.022±.009 | | |
| *Escherichia-Shigella* | 0.141±0.090 | | 0.580±0.330 | 0.624±0.505 | | 0.782±0.709 | | |
| *Eubacterium* | 0.037±0.037 | | 0.001±0.001 | 0.070±0.056 | | 0.004±0.004 | | |
| *Faecalibacterium* | 0.072±0.065 | | 0.202±0.105 | 0.473±0.294 | | 0.219±0.084 | | |
| *Flavonifractor* | 0.078±0.040 | | 0.011±0.006 | 0.000±0.000 | | 0.000±0.000 | | |
| *Fusobacterium* | 2.273±1.230 | | 7.628±5.487 | 0.220±0.131 | | 0.375±0.237 | | |
| *Haemophilus* | 0.086±0.067 | | 0.175±0.104 | 0.002±0.001 | | 0.002±0.002 | | |
| *Hydrogenoanaerobacterium* | 0.164±0.063 | | 0.030±0.009 | 0.006±0.003 | | 0.031±0.031 | | |
| Incertae_Sedis | 3.188±0.948 | | 5.462±1.448 | 2.620±0.362 | | 2.614±0.515 | | |
| *Intestinimonas* | 0.383±0.321 | | 0.658±0.331 | 0.129±0.061 | | 1.057±0.955 | | |
| *Lactobacillus* | 6.609±1.669 | | 11.00±4.417 | 17.53±9.344 | | 13.74±5.672 | | |
| *Marvinbryantia* | 0.000±0.000 | | 0.006±0.004 | 0.048±0.018 | | 0.054±0.018 | | |
| *Megasphaera* | 0.000±0.000 | | 0.001±0.001 | 0.096±0.096 | | 0.068±0.063 | | |
| *Mogibacterium* | 0.000±0.000 | | 0.026±0.022 | 0.135±0.047 | | 0.107±0.061 | | |
| norank Bacteroidales p-2534-18B5_gut _group | 0.009±0.009 | | 0.898±0.897 | 0.010±0.008 | | 0.077±0.077 | | |
| norank Bacteroidales S24-7 | 2.190±1.607 | | 7.105±3.751 | 9.708±4.642 | | 8.657±3.616 | | |
| norank Clostridiales vadinBB60 | 0.253±0.235 | | 0.062±0.042 | 0.028±0.025 | | 0.011±0.007 | | |
| norank Gastranaerophilales | 0.144±0.144 | | 0.000±0.000 | 0.078±0.075 | | 0.016±0.012 | | |
| norank Mollicutes RF9 | 0.000±0.000 | | 0.023±0.023 | 0.154±0.102 | | 0.083±0.076 | | |
| *Odoribacter* | 0.008±0.008 | | 0.438±0.273 | 0.015±0.013 | | 0.035±0.031 | | |
| *Oribacterium* | 0.000±0.000 | | 0.003±0.003 | 0.043±0.032 | | 0.051±0.040 | | |
| *Oscillibacter* | 0.198±0.126 | | 0.027±0.010 | 0.075±0.042 | | 0.066±0.032 | | |
| *Parabacteroides* | 1.690±1.330 | | 0.319±0.042 | 0.556±0.480 | | 0.370±0.206 | | |
| *Pasteurella* | 0.327±0.208 | | 1.720±1.438 | 0.002±0.001 | | 0.005±0.004 | | |
| *Peptococcus* | 0.004±0.004 | | 0.017±0.012 | 0.078±0.046 | | 0.032±0.019 | | |
| *Peptostreptococcus* | 0.024±0.008 | | 0.069±0.030 | 0.029±0.010 | | 0.003±0.002* | | |
| *Phascolarctobacterium* | 2.107±1.038 | | 3.679±0.939 | 3.058±1.647 | | 2.221±0.939 | | |
| *Prevotella* | 5.343±5.134 | | 5.543±2.579 | 0.479±0.285 | | 2.579±1.153 | | |
| *Pseudoflavonifractor* | 1.143±1.073 | | 0.042±0.020 | 0.009±0.006 | | 0.002±0.001 | | |
| *Pyramidobacter* | 0.000±0.000 | | 0.000±0.000 | 0.675±0.674 | | 0.203±0.197 | | |
| RC9_gut_group | 1.110±0.934 | | 0.664±0.652 | 0.424±0.231 | | 0.592±0.429 | | |
| *Roseburia* | 0.018±0.018 | | 1.130±0.679 | 1.399±0.820 | | 0.170±0.057 | | |
| *Ruminococcus* | 0.095±0.030 | | 0.162±0.053 | 0.322±0.186 | | 0.597±0.216 | | |
| *Spirochaeta* | 0.985±0.979 | | 0.018±0.017 | 0.035±0.033 | | 0.021±0.017 | | |
| *Streptococcus* | 0.682±0.393 | | 1.839±0.812 | 0.575±0.218 | | 1.676±1.264 | | |
| *Subdoligranulum* | 0.272±0.085 | | 1.064±0.326 | 4.156±1.731 | | 1.786±1.002 | | |
| *Succiniclasticum* | 0.000±0.000 | | 2.011±1.400 | 0.000±0.000 | | 0.000±0.000 | | |
| *Synergistes* | 0.000±0.000 | | 0.000±0.000 | 0.300±0.299 | | 0.018±0.013 | | |
| uncultured Christensenellaceae | 0.009±0.005 | | 0.024±0.010 | 0.523±0.402 | | 0.336±0.124 | | |
| uncultured Clostridiales Family_XIII | 0.368±0.203 | | 1.194±0.867 | 0.787±0.261 | | 1.774±1.164 | | |
| uncultured Erysipelotrichaceae | 0.221±0.216 | | 0.526±0.507 | 0.755±0.331 | | 2.701±1.235 | | |
| uncultured Lachnospiraceae | 0.097±0.073 | | 0.205±0.114 | 0.035±0.025 | | 0.026±0.009 | | |
| uncultured Prevotellaceae | 6.348±6.216 | | 2.409±0.642 | 4.267±3.701 | | 2.501±0.898 | | |
| uncultured Ruminococcaceae | 4.237±1.034 | | 6.524±0.894 | 33.61±15.91 | | 21.54±10.14 | | |
| *Veillonella* | 0.523±0.118 | | 1.674±0.422 | 0.144±0.053 | | 0.065±0.032 | | |
| unclassified Bacteroidetes | 0.071±0.069 | | 0.032±0.028 | 0.007±0.004 | | 0.018±0.011 | | |
| unclassified Clostridiaceae_1 | 0.107±0.063 | | 0.043±0.027 | 0.001±0.001 | | 0.000±0.000 | | |
| unclassified Clostridiales | 0.020±0.013 | | 0.035±0.027 | 0.017±0.009 | | 0.079±0.066 | | |
| unclassified Clostridiales Family_XIII | 0.000±0.000 | | 0.055±0.055 | 0.000±0.000 | | 0.004±0.004 | | |
| unclassified Lachnospiraceae | 0.105±0.042 | | 0.706±0.373 | 0.751±0.314 | | 3.242±1.889 | | |
| unclassified Lactobacillales | 0.059±0.042 | | 0.073±0.023 | 0.011±0.004 | | 0.036±0.021 | | |
| unclassified Prevotellaceae | 0.041±0.041 | | 0.004±0.003 | 0.096±0.074 | | 0.030±0.022 | | |
| unclassified Ruminococcaceae | 2.172±1.136 | | 1.168±0.128 | 1.739±0.558 | | 0.813±0.225 | | |

## ^1^Genera with relative abundances higher than 0.05% within total bacteria were sorted and showed in the table.

## * means the significantly difference (*P* < 0.05) between SB group and CO group.

## ** means the significantly difference (*P* < 0.01) between SB group and CO group.
