# Supplementary material for: Effects of Early Intervention with Sodium Butyrate on Gut Microbiota and the Expression of Inflammatory Cytokines in Neonatal Piglets
Source: PLoS One. 2016 Sep 9;11(9):e0162461. doi: 10.1371/journal.pone.0162461 (PMC5017769; doi:10.1371/journal.pone.0162461)
Supplement: S11 Table — (DOC) [file pone.0162461.s013.doc]

S11 Table. Relative abundances of microbial OTUs (percentage) that were affected by the sodium butyrate treatment in the ileum of pigs (n=5).^1^

| OTU Names | 8d |  | 21d |  | Annotation^2^ |
| --- | --- | --- | --- | --- | --- |
|  | CO | SB | CO | SB |  |
| OTU655 | 15.58±6.054 | 19.22±10.31 | 27.69±10.17 | 24.05±5.641 | g__*Lactobacillus* |
| OTU792 | 35.82±9.834 | 19.17±7.920 | 24.20±7.983 | 22.03±6.500 | g__*Lactobacillus* |
| OTU379 | 2.211±0.569 | 2.508±0.972 | 4.380±2.332 | 21.77±11.14 | s__*Streptococcus*_*gallolyticus*_subsp._*macedonicus* |
| OTU431 | 0.999±0.771 | 3.000±2.085 | 0.246±0.192 | 4.235±1.944** | f__Peptostreptococcaceae |
| OTU573 | 1.718±0.739 | 4.032±2.789 | 4.409±3.028 | 3.046±1.482 | g__*Streptococcus* |
| OTU581 | 6.075±3.304 | 3.905±1.718 | 3.746±1.490 | 2.576±0.975 | g__*Veillonella* |
| OTU806 | 0.266±0.120 | 8.476±8.075 | 1.926±1.018 | 2.296±0.953 | g__*Lactobacillus* |
| OTU13 | 6.201±3.117 | 1.516±0.518 | 11.34±7.423 | 2.192±0.932 | g__*Lactobacillus* |
| OTU235 | 1.140±1.120 | 0.307±0.166 | 0.428±0.253 | 1.400±0.703 | s__*Lactobacillus*_*coleohominis* |
| OTU92 | 0.000±0.000 | 0.011±0.011 | 0.460±0.202 | 1.062±0.507 | g__*Streptococcus* |
| OTU527 | 0.606±0.294 | 0.145±0.133 | 0.606±0.407 | 1.041±0.436 | g__*Lactobacillus* |
| OTU789 | 0.000±0.000 | 0.004±0.004 | 0.683±0.319 | 0.934±0.637 | s__*Actinomyces*_*denticolens* |
| OTU76 | 0.058±0.031 | 0.197±0.139 | 0.064±0.040 | 0.810±0.487 | s__[*Clostridium*]_*glycolicum* |
| OTU535 | 0.460±0.239 | 0.130±0.120 | 0.509±0.316 | 0.726±0.305 | g__*Lactobacillus* |
| OTU692 | 0.425±0.135 | 0.669±0.453 | 0.778±0.525 | 0.631±0.174 | g__*Gemella* |
| OTU832 | 0.063±0.047 | 0.056±0.025 | 0.107±0.047 | 0.608±0.393 | f__Peptostreptococcaceae |
| OTU424 | 1.091±0.466 | 0.883±0.539 | 0.452±0.263 | 0.515±0.220 | g__*Streptococcus* |
| OTU699 | 0.048±0.032 | 0.139±0.060 | 0.097±0.054 | 0.496±0.247 | s__*Streptococcus*_*orisratti* |
| OTU419 | 0.217±0.075 | 0.202±0.163 | 0.740±0.465 | 0.495±0.247 | s__TM7_phylum_sp._oral_clone_FR058 |
| OTU625 | 0.421±0.176 | 0.263±0.160 | 0.765±0.330 | 0.460±0.108 | g__*Peptostreptococcus* |
| OTU266 | 0.174±0.068 | 0.095±0.061 | 0.099±0.056 | 0.432±0.239 | g__*Clostridium*_*sensu*_*stricto*_1 |
| OTU441 | 0.049±0.023 | 0.088±0.027 | 0.072±0.025 | 0.399±0.312 | g__*Turicibacter* |
| OTU622 | 0.046±0.014 | 0.137±0.090 | 0.465±0.404 | 0.388±0.150 | s__*Corynebacterium*_*testudinoris* |
| OTU125 | 0.089±0.051 | 0.099±0.081 | 0.066±0.043 | 0.340±0.144 | s__*Corynebacterium*_*freneyi* |
| OTU820 | 2.194±0.898 | 6.779±4.555 | 1.107±0.928 | 0.290±0.191 | s__*Lactobacillus*_*johnsonii* |
| OTU145 | 0.017±0.008 | 0.004±0.002 | 0.044±0.038 | 0.279±0.0698* | o__Lactobacillales |
| OTU301 | 0.340±0.178 | 2.479±2.308 | 0.297±0.268 | 0.246±0.243 | s__*Actinobacillus*_*porcinus* |
| OTU391 | 0.103±0.042 | 0.195±0.150 | 0.279±0.125 | 0.234±0.066 | f__Lachnospiraceae |
| OTU763 | 0.031±0.017 | 0.003±0.003 | 0.014±0.009 | 0.222±0.086 | o__Lactobacillales |
| OTU842 | 0.072±0.030 | 0.016±0.016 | 0.057±0.041 | 0.213±0.105 | g__*Lactobacillus* |
| OTU211 | 10.06±6.666 | 5.228±4.684 | 0.560±0.332 | 0.211±0.131 | g__*Veillonella* |
| OTU738 | 0.025±0.016 | 0.021±0.009 | 0.258±0.149 | 0.184±0.048 | g__*Streptococcus* |
| OTU474 | 0.069±0.020 | 0.203±0.171 | 0.193±0.132 | 0.175±0.054 | s__*Globicatella*_sp._canine_oral_taxon_218 |
| OTU47 | 0.060±0.015 | 0.162±0.104 | 0.167±0.073 | 0.162±0.028 | g__*Rothia* |
| OTU34 | 0.132±0.035 | 1.353±0.742 | 0.303±0.165 | 0.154±0.047 | s__*Pseudomonas*_*vranovensis* |
| OTU558 | 0.126±0.062 | 0.217±0.141 | 0.066±0.023 | 0.152±0.035 | s__*Streptococcus*_*thoraltensis*_DSM_12221 |
| OTU202 | 3.243±2.398 | 1.417±0.740 | 0.446±0.233 | 0.142±0.049 | g__*Fusobacterium* |
| OTU460 | 0.049±0.023 | 0.108±0.095 | 0.474±0.381 | 0.142±0.049 | o__Lactobacillales |
| OTU99 | 0.113±0.046 | 0.119±0.090 | 0.310±0.255 | 0.142±0.049 | g__*Streptococcus* |
| OTU141 | 0.017±0.015 | 0.003±0.003 | 0.285±0.250 | 0.122±0.058 | s__*Streptococcus*_*plurextorum* |
| OTU245 | 0.140±0.118 | 0.050±0.017 | 0.032±0.018 | 0.121±0.083 | g__*Arcanobacterium* |
| OTU551 | 0.017±0.011 | 0.243±0.233 | 0.540±0.374 | 0.119±0.057 | s__*Lactobacillus*_*mucosae* |
| OTU439 | 0.078±0.032 | 0.047±0.023 | 1.396±1.234 | 0.110±0.025 | g__*Actinomyces* |
| OTU217 | 0.051±0.027 | 0.011±0.008 | 0.057±0.021 | 0.106±0.048 | g__*Howardella* |
| OTU839 | 0.008±0.003 | 0.009±0.003 | 0.165±0.084 | 0.102±0.063 | g__*Veillonella* |
| OTU542 | 0.004±0.003 | 0.004±0.004 | 0.363±0.294 | 0.101±0.062 | f__Lachnospiraceae |
| OTU229 | 0.044±0.014 | 0.144±0.113 | 0.031±0.016 | 0.097±0.056 | s__*Streptococcus*_*pluranimalium* |
| OTU534 | 0.000±0.000 | 0.020±0.014 | 0.010±0.006 | 0.083±0.046 | f__Family_XIII |
| OTU813 | 0.000±0.000 | 0.021±0.021 | 0.124±0.071 | 0.083±0.050 | g__*Fusobacterium* |
| OTU646 | 0.000±0.000 | 0.002±0.002 | 0.050±0.027 | 0.077±0.023 | g__*Johnsonella* |
| OTU664 | 0.022±0.020 | 0.027±0.020 | 0.083±0.070 | 0.075±0.023 | g__*Corynebacterium* |
| OTU14 | 0.023±0.023 | 0.064±0.056 | 0.323±0.214 | 0.071±0.042 | f__Enterococcaceae |
| OTU831 | 0.061±0.047 | 0.031±0.010 | 0.137±0.078 | 0.065±0.039 | g__*Arcanobacterium* |
| OTU310 | 0.129±0.080 | 1.907±1.693 | 0.156±0.065 | 0.064±0.053 | s__*Actinobacillus*_*minor* |
| OTU174 | 0.050±0.031 | 0.021±0.014 | 0.031±0.020 | 0.063±0.030 | g__*Helcococcus* |
| OTU316 | 0.003±0.002 | 0.003±0.003 | 0.005±0.003 | 0.063±0.041 | g__*Lactobacillus* |
| OTU366 | 0.118±0.056 | 0.057±0.035 | 0.050±0.023 | 0.063±0.023 | g__*Corynebacterium* |
| OTU73 | 0.003±0.003 | 0.007±0.002 | 0.016±0.007 | 0.063±0.058 | f__Erysipelotrichaceae |
| OTU173 | 0.070±0.054 | 0.030±0.021 | 0.064±0.032 | 0.056±0.036 | s__*Streptococcus*_*minor* |
| OTU850 | 0.039±0.039 | 0.223±0.222 | 0.303±0.298 | 0.054±0.033 | g__*Corynebacterium* |
| OTU315 | 0.005±0.003 | 0.003±0.003 | 0.004±0.003 | 0.053±0.034 | g__*Lactobacillus* |
| OTU463 | 0.178±0.099 | 0.428±0.233 | 0.100±0.066 | 0.048±0.007 | g__*Escherichia*-*Shigella* |
| OTU46 | 2.772±2.769 | 0.011±0.009 | 0.016±0.009 | 0.045±0.045 | s__*Actinobacillus*_*rossii* |
| OTU9 | 0.010±0.006 | 0.017±0.017 | 0.075±0.038 | 0.042±0.019 | f__Lachnospiraceae |
| OTU365 | 0.026±0.007 | 0.074±0.029 | 0.026±0.011 | 0.041±0.020 | f__Alcaligenaceae |
| OTU753 | 0.019±0.011 | 0.051±0.043 | 0.066±0.061 | 0.040±0.018 | g__*Granulicatella* |
| OTU588 | 0.002±0.002 | 0.001±0.001 | 0.051±0.027 | 0.038±0.015 | g__*Parvimonas* |
| OTU236 | 0.000±0.000 | 0.005±0.005 | 0.086±0.037 | 0.034±0.015 | s__*Clostridiales*_*bacterium*_canine_oral_taxon_100 |
| OTU533 | 0.000±0.000 | 0.000±0.000 | 0.075±0.040 | 0.034±0.018 | f__Ruminococcaceae |
| OTU381 | 0.002±0.001 | 0.041±0.022* | 0.105±0.068 | 0.034±0.031 | f__Erysipelotrichaceae |
| OTU545 | 0.013±0.008 | 0.015±0.009 | 0.087±0.079 | 0.033±0.013 | g__*Facklamia* |
| OTU787 | 0.027±0.014 | 0.027±0.014 | 0.067±0.048 | 0.031±0.007 | f__Family_XIII |
| OTU744 | 0.004±0.003 | 0.002±0.002 | 0.066±0.045 | 0.030±0.012 | g__*Leucobacter* |
| OTU841 | 0.044±0.044 | 0.054±0.037 | 0.220±0.151 | 0.024±0.016 | f__Lachnospiraceae |
| OTU561 | 0.015±0.005 | 0.030±0.022 | 0.090±0.079 | 0.020±0.009 | f__Erysipelotrichaceae |
| OTU340 | 0.175±0.125 | 0.191±0.087 | 0.024±0.012 | 0.019±0.007 | p__Candidate_division_TM7 |
| OTU756 | 0.010±0.008 | 0.061±0.057 | 0.042±0.033 | 0.019±0.011 | s__*Streptococcus*_*parauberis* |
| OTU667 | 0.004±0.003 | 0.042±0.042 | 0.202±0.202 | 0.015±0.007 | g__*Globicatella* |
| OTU192 | 1.428±1.396 | 6.107±4.583 | 0.023±0.020 | 0.012±0.008 | s__*Pasteurella*_*aerogenes* |
| OTU56 | 0.000±0.000 | 0.001±0.001 | 0.056±0.046 | 0.011±0.010 | s__*Dermabacter*_*hominis* |
| OTU178 | 0.029±0029 | 0.011±0.009 | 0.235±0.235 | 0.011±0.010 | g__*Nosocomiicoccus* |
| OTU457 | 0.275±0.153 | 0.730±0.283 | 0.030±0.019 | 0.010±0.007 | g__*Moraxella* |
| OTU10 | 0.003±0.003 | 0.051±0.051 | 0.006±0.004 | 0.010±0.010 | g__*Leptotrichia* |
| OTU55 | 0.161±0.113 | 0.035±0.028 | 0.000±0.000 | 0.010±0.007 | s__*Clostridium*_*perfringens*_NCTC_8239 |
| OTU123 | 0.007±0.003 | 0.002±0.002 | 0.053±0.050 | 0.009±0.005 | f__Aerococcaceae |
| OTU42 | 0.065±0.061 | 0.008±0.003 | 0.025±0.024 | 0.008±0.003 | f__Ruminococcaceae |
| OTU395 | 0.003±0.002 | 0.010±0.007 | 0.256±0.254 | 0.008±0.006 | g__*Anaerococcus* |
| OTU680 | 0.012±0.010 | 0.008±0.005 | 0.171±0.171 | 0.006±0.002 | g__*Ignavigranum* |
| OTU615 | 0.001±0.001 | 0.055±0.047 | 0.006±0.005 | 0.006±0.006 | s__*Moraxella*_*osloensis* |
| OTU860 | 0.004±0.004 | 0.003±0.002 | 0.069±0.068 | 0.005±0.005 | s__*Jeotgalicoccus*_sp._M3T9B12 |
| OTU722 | 0.109±0.079 | 1.443±0.864 | 0.057±0.042 | 0.004±0.002 | g__*Haemophilus* |
| OTU32 | 0.000±0.000 | 0.364±0.360 | 0.011±0.011 | 0.004±0.002 | s__*Clostridium*_sp._ND2 |
| OTU788 | 0.009±0.003 | 0.056±0.033 | 0.010±0.003 | 0.004±0.002 | g__*Pseudomonas* |
| OTU729 | 0.070±0.047 | 0.008±0.004 | 0.002±0.001 | 0.003±0.003 | g__*Phascolarctobacterium* |
| OTU786 | 0.005±0.003 | 0.014±0.014 | 0.053±0.053 | 0.002±0.002 | f__Family_XI |
| OTU248 | 0.056±0.014 | 0.019±0.013 | 0.001±0.001 | 0.0010±0.001 | g__*Bacteroides* |
| OTU377 | 0.061±0.060 | 0.012±0.012 | 0.003±0.003 | 0.001±0.001 | f__Pasteurellaceae |
| OTU425 | 0.000±0.000 | 0.000±0.000 | 0.140±0.140 | 0.001±0.001 | f__Ruminococcaceae |
| OTU863 | 0.003±0.003 | 0.000±0.000 | 0.052±0.052 | 0.001±0.001 | g__*Solobacterium* |
| OTU866 | 0.106±0.105 | 0.009±0.008 | 0.011±0.011 | 0.001±0.001 | f__Lachnospiraceae |
| OTU22 | 0.006±0.003 | 0.156±0.073 | 0.000±0.000 | 0.000±0.000 | s__*Bergeyella*_*zoohelcum* |
| OTU162 | 0.056±0.018 | 0.034±0.025 | 0.000±0.000 | 0.000±0.000 | f__Prevotellaceae |
| OTU243 | 0.297±0.280 | 0.323±0.280 | 0.198±1.900 | 0.000±0.000* | g__*Sarcina* |
| OTU333 | 0.059±0.023 | 0.023±0.017 | 0.000±0.000 | 0.000±0.000 | o__Bacteroidales |
| OTU475 | 0.064±0.051 | 0.783±0.768 | 0.002±0.002 | 0.000±0.000 | s__Fusobacterium_mortiferum |
| OTU653 | 0.108±0.031 | 0.059±0.038 | 0.003±0.002 | 0.000±0.000 | g__*Megamonas* |
| OTU734 | 0.000±0.000 | 0.000±0.000 | 0.051±0.051 | 0.000±0.000 | g__*Helcococcus* |
| OTU822 | 0.083±0.027 | 0.037±0.024 | 0.000±0.000 | 0.000±0.000 | g__*Bacteroides* |

## ^1^OTUs with relative abundances higher than 0.05% within total bacteria were sorted and showed in the table.

^2^The consensus sequence of each OTU was annotated to the closest lineage using MOTHUR program against the SILVA 16S rRNA reference database. s = species; g = genus; f = family; o = order**.**

## * means the significantly difference (*P* < 0.05) between SB group and CO group.

## ** means the significantly difference (*P* < 0.01) between SB group and CO group.
