# Supplementary material for: Effects of Early Intervention with Sodium Butyrate on Gut Microbiota and the Expression of Inflammatory Cytokines in Neonatal Piglets
Source: PLoS One. 2016 Sep 9;11(9):e0162461. doi: 10.1371/journal.pone.0162461 (PMC5017769; doi:10.1371/journal.pone.0162461)
Supplement: S12 Table — (DOC) [file pone.0162461.s014.doc]

S12 Table. Relative abundances of microbial OTUs (percentage) that were affected by the sodium butyrate treatment in the colon of piglets (n=5).^1^

| **OTU Names** | **8d** | |  | | **21d** |  | **Annotation**^2^ |
| --- | --- | --- | --- | --- | --- | --- | --- |
|  | **CO** | **SB** | | **CO** | | **SB** |  |
| OTU681 | 0.000±0.000 | 0.103±0.100 | | 14.93±14.45 | | 14.29±14.01 | f__Ruminococcaceae |
| OTU807 | 4.057±2.133 | 3.732±1.601 | | 2.119±0.930 | | 11.48±6.296 | g__*Anaerotruncus* |
| OTU655 | 0.613±0.363 | 2.641±1.712 | | 10.10±5.759 | | 10.11±3.889 | g__*Lactobacillus* |
| OTU743 | 0.418±0.418 | 0.210±0.152 | | 0.047±0.039 | | 9.235±5.902 | s__*Bacteroides_massiliensis* |
| OTU584 | 0.000±0.000 | 0.033±0.033 | | 2.183±1.239 | | 3.351±1.835 | f__S24-7 |
| OTU29 | 0.218±0.217 | 0.510±0.508 | | 0.345±0.186 | | 2.542±1.144 | f__Erysipelotrichaceae |
| OTU525 | 0.403±0.375 | 3.031±2.832 | | 5.262±3.675 | | 2.385±1.654 | f__S24-7 |
| OTU729 | 1.596±1.000 | 2.652±0.992 | | 3.009±1.663 | | 2.222±0.939 | g__*Phascolarctobacterium* |
| OTU792 | 3.395±1.312 | 3.654±1.561 | | 4.722±2.264 | | 1.974±1.077 | g__*Lactobacillus* |
| OTU755 | 0.000±0.000 | 0.004±0.004 | | 0.014±0.010 | | 1.868±1.764 | f__Ruminococcaceae |
| OTU740 | 0.362±0.204 | 1.170±0.867 | | 0.636±0.256 | | 1.720±1.175 | f__Family_XIII |
| OTU325 | 0.003±0.003 | 0.005±0.004 | | 0.059±0.049 | | 1.673±1.667 | f__Lachnospiraceae |
| OTU379 | 0.445±0.247 | 1.446±0.727 | | 0.357±0.177 | | 1.544±1.233 | s__*Streptococcus_gallolyticus_subsp._macedonicus* |
| OTU294 | 6.230±6.230 | 0.003±0.002 | | 0.554±0.493 | | 1.468±0.891 | f__Prevotellaceae |
| OTU705 | 0.203±0.083 | 0.950±0.343 | | 3.706±1.700 | | 1.330±0.891 | g__*Subdoligranulum* |
| OTU135 | 0.000±0.000 | 0.006±0.006 | | 0.040±0.036 | | 1.150±1.147 | g__*Prevotella* |
| OTU2 | 0.464±0.319 | 0.367±0.108 | | 0.097±0.053 | | 1.141±0.638 | g__*Anaerotruncus* |
| OTU159 | 0.019±0.011 | 0.545±0.354 | | 0.033±0.019 | | 1.010±0.957 | g__*Intestinimonas* |
| OTU693 | 1.457±1.003 | 0.444±0.140 | | 9.633±8.670 | | 1.000±0.648 | f__Ruminococcaceae |
| OTU238 | 0.887±0.432 | 2.886±1.511 | | 0.748±0.273 | | 0.897±0.278 | s__human_gut_*metagenome* |
| OTU704 | 0.000±0.000 | 0.383±0.363 | | 2.399±1.135 | | 0.888±0.269 | f__Ruminococcaceae |
| OTU205 | 0.263±0.163 | 0.111±0.055 | | 0.125±0.051 | | 0.859±0.532 | s__*Ruminococcus*_sp._5_1_39BFAA |
| OTU848 | 19.18±9.397 | 6.836±2.499 | | 0.165±0.149 | | 0.805±0.777 | g__*Bacteroides* |
| OTU463 | 0.141±0.090 | 0.580±0.330 | | 0.624±0.505 | | 0.782±0.709 | g__*Escherichia*-*Shigella* |
| OTU416 | 0.093±0.047 | 3.578±2.131 | | 0.094±0.067 | | 0.773±0.761 | s__*Bacteroides*_*plebeius* |
| OTU749 | 0.043±0.024 | 0.241±0.154 | | 0.476±0.260 | | 0.750±0.351 | f__Erysipelotrichaceae |
| OTU554 | 0.019±0.014 | 0.042±0.042 | | 0.031±0.014 | | 0.723±0.712 | g__*Prevotella* |
| OTU175 | 0.147±0.145 | 5.225±2.636* | | 0.406±0.244 | | 0.702±0.631 | s__*Prevotella*_sp._DJF_LS16 |
| OTU589 | 0.000±0.000 | 0.011±0.010 | | 0.471±0.455 | | 0.681±0.448 | f__Ruminococcaceae |
| OTU170 | 0.000±0.000 | 0.001±0.001 | | 0.359±0.320 | | 0.644±0.586 | g__*Alloprevotella* |
| OTU833 | 0.006±0.004 | 0.166±0.164 | | 1.576±1.266 | | 0.625±0.390 | g__*Bacteroides* |
| OTU464 | 0.0040.004 | 0.030±0.030 | | 0.048±0.030 | | 0.564±0.463 | f__S24-7 |
| OTU560 | 0.004±0.002 | 0.040±0.025 | | 0.014±0.010 | | 0.487±0.468 | f__Prevotellaceae |
| OTU527 | 0.144±0.055 | 0.304±0.198 | | 0.723±0.499 | | 0.455±0.279 | g__*Lactobacillus* |
| OTU461 | 0.000±0.000 | 0.000±0.000 | | 0.060±0.038 | | 0.453±0.339 | f__Ruminococcaceae |
| OTU67 | 0.093±0.032 | 0.161±0.053 | | 0.319±0.186 | | 0.452±0.236 | s__*bacterium*_ic1277 |
| OTU754 | 0.003±0.002 | 0.012±0.005 | | 0.216±0.105 | | 0.415±0.165 | g__*Subdoligranulum* |
| OTU689 | 0.229±0.084 | 0.263±0.077 | | 0.720±0.364 | | 0.403±0.178 | f__Ruminococcaceae |
| OTU137 | 0.029±0.013 | 0.163±0.061 | | 0.191±0.066 | | 0.394±0.195 | f__Lachnospiraceae |
| OTU576 | 0.003±0.003 | 0.442±0.285 | | 0.299±0.282 | | 0.392±0.302 | f__Lachnospiraceae |
| OTU431 | 0.199±0.133 | 0.702±0.277 | | 0.331±0.191 | | 0.378±0.096 | f__Peptostreptococcaceae |
| OTU823 | 0.611±0.311 | 1.420±0.483 | | 0.402±0.159 | | 0.354±0.234 | f__Ruminococcaceae |
| OTU286 | 0.000±0.000 | 0.001±0.001 | | 0.112±0.112 | | 0.352±0.351 | f__S24-7 |
| OTU507 | 0.006±0.005 | 0.073±0.071 | | 2.022±1.475 | | 0.352±0.153 | g__*Collinsella* |
| OTU799 | 0.155±0.155 | 0.039±0.027 | | 0.092±0.068 | | 0.348±0.154 | f__S24-7 |
| OTU80 | 0.035±0.019 | 0.090±0.062 | | 0.118±0.069 | | 0.346±0.079* | f__S24-7 |
| OTU13 | 1.620±1.206 | 1.140±0.563 | | 0.866±0.546 | | 0.340±0.114 | g__*Lactobacillus* |
| OTU535 | 0.112±0.045 | 0.227±0.143 | | 0.472±0.314 | | 0.287±0.175 | g__*Lactobacillus* |
| OTU70 | 0.000±0.000 | 0.024±0.024 | | 0.055±0.039 | | 0.274±0.236 | f__S24-7 |
| OTU261 | 0.464±0.377 | 1.083±0.749 | | 0.337±0.168 | | 0.270±0.084 | f__Lachnospiraceae |
| OTU235 | 0.063±0.061 | 0.025±0.019 | | 0.202±0.177 | | 0.268±0.172 | s__*Lactobacillus_coleohominis* |
| OTU191 | 0.000±0.000 | 0.000±0.000 | | 0.095±0.064 | | 0.263±0.263 | g__RC9_gut_group |
| OTU432 | 0.005±0.002 | 0.012±0.009 | | 0.308±0.119 | | 0.262±0.112 | f__Lachnospiraceae |
| OTU725 | 0.010±0.007 | 0.007±0.003 | | 0.057±0.029 | | 0.261±0.105 | g__*Blautia* |
| OTU193 | 0.000±0.000 | 0.000±0.000 | | 0.001±0.001 | | 0.253±0.251 | f__Lachnospiraceae |
| OTU376 | 0.000±0.000 | 0.001±0.001 | | 0.000±0.000 | | 0.241±0.241 | f__S24-7 |
| OTU843 | 0.001±0.001 | 0.000±0.000 | | 0.803±0.760 | | 0.238±0.106 | f__Ruminococcaceae |
| OTU509 | 0.005±0.005 | 0.067±0.063 | | 0.309±0.282 | | 0.236±0.114 | f__Prevotellaceae |
| OTU266 | 0.107±0.091 | 0.297±0.182 | | 0.057±0.042 | | 0.219±0.115 | g__*Clostridium*_*sensu*_*stricto*_1 |
| OTU835 | 0.000±0.000 | 0.000±0.000 | | 0.675±0.674 | | 0.203±0.197 | g__*Pyramidobacter* |
| OTU824 | 0.968±0.806 | 0.609±0.597 | | 0.164±0.150 | | 0.199±0.134 | g__RC9_gut_group |
| OTU716 | 0.094±0.051 | 0.190±0.138 | | 0.155±0.067 | | 0.193±0.087 | s__*Blautia*_*glucerasea* |
| OTU219 | 0.036±0.018 | 0.070±0.045 | | 0.102±0.051 | | 0.177±0.098 | f__Lachnospiraceae |
| OTU202 | 0.845±0.531 | 0.515±0.311 | | 0.031±0.020 | | 0.171±0.152 | g__*Fusobacterium* |
| OTU314 | 0.014±0.012 | 0.135±0.079 | | 0.371±0.234 | | 0.160±0.076 | g__*Faecalibacterium* |
| OTU518 | 0.251±0.087 | 0.385±0.094 | | 0.242±0.073 | | 0.158±0.101 | g__Incertae_Sedis |
| OTU358 | 0.657±0.370 | 0.442±0.088 | | 0.470±0.273 | | 0.155±0.053 | f__Ruminococcaceae |
| OTU126 | 1.161±0.437 | 2.491±1.146 | | 0.111±0.050 | | 0.147±0.080 | g__*Alistipes* |
| OTU31 | 0.002±0.002 | 0.000±0.000 | | 0.355±0.301 | | 0.146±0.116 | f__Erysipelotrichaceae |
| OTU842 | 0.019±0.009 | 0.065±0.040 | | 0.149±0.120 | | 0.140±0.080 | g__*Lactobacillus* |
| OTU467 | 0.000±0.000 | 0.053±0.053 | | 0.948±0.610 | | 0.139±0.076 | f__Ruminococcaceae |
| OTU691 | 0.823±0.762 | 0.070±0.014 | | 0.003±0.002 | | 0.138±0.073 | s__*Parabacteroides*_*merdae* |
| OTU230 | 0.000±0.000 | 0.001±0.001 | | 0.013±0.005 | | 0.138±0.120 | f__Lachnospiraceae |
| OTU838 | 0.548±0.295 | 0.154±0.075 | | 0.399±0.397 | | 0.137±0.131 | s__*Parabacteroides*_*distasonis*_ATCC_8503 |
| OTU475 | 1.277±0.863 | 7.050±5.195 | | 0.165±0.128 | | 0.136±0.105 | s__*Fusobacterium*_*mortiferum* |
| OTU323 | 0.038±0.030 | 0.065±0.033 | | 0.342±0.273 | | 0.135±0.058 | f__Ruminococcaceae |
| OTU832 | 0.005±0.004 | 0.007±0.004 | | 0.040±0.016 | | 0.133±0.034 | f__Peptostreptococcaceae |
| OTU117 | 0.002±0.002 | 0.006±0.004 | | 0.132±0.124 | | 0.131±0.084 | f__S24-7 |
| OTU765 | 0.018±0.018 | 1.130±0.679 | | 1.209±0.694 | | 0.130±0.062 | g__*Roseburia* |
| OTU84 | 0.124±0.057 | 0.122±0.070 | | 0.150±0.109 | | 0.112±0.045 | s__*Clostridiales*_*bacterium*_canine_oral_taxon_085 |
| OTU131 | 1.189±1.189 | 0.003±0.003 | | 0.016±0.013 | | 0.108±0.073 | f__S24-7 |
| OTU6 | 0.000±0.000 | 0.026±0.022 | | 0.135±0.047 | | 0.107±0.061 | g__*Mogibacterium* |
| OTU697 | 0.041±0.037 | 0.000±0.000 | | 0.022±0.009 | | 0.106±0.090 | f__Prevotellaceae |
| OTU694 | 0.004±0.004 | 0.012±0.006 | | 0.003±0.002 | | 0.104±0.067 | g__*Alloprevotella* |
| OTU481 | 0.028±0.021 | 0.024±0.010 | | 0.261±0.261 | | 0.104±0.104 | f__Ruminococcaceae |
| OTU575 | 0.069±0.036 | 3.102±2.766 | | 0.010±0.005 | | 0.102±0.102 | f__S24-7 |
| OTU352 | 0.000±0.000 | 0.001±0.001 | | 0.050±0.045 | | 0.102±0.079 | f__Christensenellaceae |
| OTU157 | 0.019±0.012 | 0.026±0.019 | | 0.071±0.037 | | 0.095±0.026 | f__Ruminococcaceae |
| OTU43 | 0.076±0.023 | 0.144±0.068 | | 0.072±0.067 | | 0.095±0.070 | s__*Desulfovibrio*_piger |
| OTU220 | 0.001±0.001 | 0.390±0.232 | | 0.122±0.057 | | 0.094±0.080 | g__*Alloprevotella* |
| OTU306 | 0.001±0.001 | 0.000±0.000 | | 0.007±0.007 | | 0.094±0.090 | f__Lachnospiraceae |
| OTU223 | 0.014±0.009 | 0.445±0.445 | | 0.010±0.005 | | 0.091±0.048 | f__S24-7 |
| OTU423 | 0.537±0.386 | 0.085±0.064 | | 0.005±0.003 | | 0.090±0.085 | g__*Alistipes* |
| OTU166 | 0.000±0.000 | 0.000±0.000 | | 0.096±0.056 | | 0.086±0.038 | f__Christensenellaceae |
| OTU392 | 0.026±0.016 | 0.022±0.008 | | 0.028±0.017 | | 0.084±0.024 | f__Family_XIII |
| OTU375 | 7.097±7.096 | 0.087±0.084 | | 0.022±0.009 | | 0.083±0.063 | g__dgA-11_gut_group |
| OTU337 | 0.134±0.083 | 0.915±0.779 | | 0.133±0.069 | | 0.082±0.078 | f__Lachnospiraceae |
| OTU169 | 0.026±0.023 | 0.310±0.139* | | 0.008±0.008 | | 0.082±0.064 | g__*Bacteroides* |
| OTU92 | 0.000±0.000 | 0.001±0.001 | | 0.056±0.025 | | 0.081±0.057 | g__*Streptococcus* |
| OTU636 | 0.000±0.000 | 0.000±0.000 | | 0.073±0.048 | | 0.079±0.061 | f__Ruminococcaceae |
| OTU295 | 0.006±0.004 | 0.022±0.012 | | 0.020±0.009 | | 0.078±0.034 | f__Ruminococcaceae |
| OTU591 | 0.009±0.009 | 0.898±0.897 | | 0.010±0.008 | | 0.077±0.077 | f__p-2534-18B5_gut_group |
| OTU789 | 0.000±0.000 | 0.000±0.000 | | 0.075±0.034 | | 0.075±0.044 | s__*Actinomyces_denticolens* |
| OTU326 | 0.000±0.000 | 0.019±0.019 | | 0.016±0.005 | | 0.073±0.068 | f__Prevotellaceae |
| OTU494 | 0.000±0.000 | 0.000±0.000 | | 0.000±0.000 | | 0.071±0.071 | g__*Ruminococcus* |
| OTU806 | 0.113±0.063 | 0.682±0.548 | | 0.134±0.076 | | 0.070±0.052 | g__*Lactobacillus* |
| OTU661 | 0.000±0.000 | 0.000±0.000 | | 0.096±0.096 | | 0.068±0.063 | s__*Megasphaera*_*elsdenii*_DSM_20460 |
| OTU821 | 0.045±0.026 | 0.103±0.035 | | 0.111±0.048 | | 0.068±0.027 | g__*Coprococcus* |
| OTU834 | 0.058±0.022 | 0.050±0.026 | | 0.040±0.016 | | 0.067±0.026 | g__*Anaerotruncus* |
| OTU727 | 0.000±0.000 | 0.062±0.061 | | 0.023±0.019 | | 0.066±0.038 | g__*Alloprevotella* |
| OTU428 | 0.046±0.036 | 0.086±0.034 | | 0.004±0.004 | | 0.065±0.036 | g__*Parabacteroides* |
| OTU797 | 0.002±0.002 | 0.022±0.016 | | 0.018±0.013 | | 0.065±0.058 | g__*Fusobacterium* |
| OTU632 | 0.000±0.000 | 0.049±0.049 | | 0.239±0.238 | | 0.064±0.055 | f__S24-7 |
| OTU91 | 0.982±0.795 | 0.151±0.038 | | 0.047±0.022 | | 0.062±0.048 | f__Ruminococcaceae |
| OTU308 | 0.000±0.000 | 0.003±0.003 | | 0.112±0.079 | | 0.062±0.030 | g__RC9_gut_group |
| OTU708 | 0.373±0.080 | 0.332±0.202 | | 0.110±0.074 | | 0.062±0.049 | s__*Clostridium*_*clostridioforme*_CM201 |
| OTU581 | 0.247±0.074 | 1.099±0.405 | | 0.085±0.033 | | 0.060±0.029 | g__*Veillonella* |
| OTU490 | 0.000±0.000 | 0.000±0.000 | | 0.000±0.000 | | 0.059±0.058 | o__Clostridiales |
| OTU95 | 0.000±0.000 | 0.003±0.003 | | 0.022±0.008 | | 0.059±0.020 | f__S24-7 |
| OTU405 | 0.056±0.054 | 0.067±0.029 | | 0.102±0.062 | | 0.059±0.023 | g__*Faecalibacterium* |
| OTU368 | 0.000±0.000 | 0.006±0.004 | | 0.048±0.018 | | 0.054±0.018 | g__*Marvinbryantia* |
| OTU703 | 0.000±0.000 | 0.001±0.001 | | 0.000±0.000 | | 0.052±0.052 | s__*Ruminococcus_bromii* |
| OTU76 | 0.005±0.002 | 0.008±0.003 | | 0.010±0.003 | | 0.052±0.019 | s__[*Clostridium*]_*glycolicum* |
| OTU60 | 0.343±0.124 | 0.921±0.217 | | 0.034±0.025 | | 0.051±0.011 | s__*Clostridium*_*scindens* |
| OTU356 | 0.064±0.030 | 0.017±0.009 | | 0.063±0.042 | | 0.051±0.029 | g__*Oscillibacter* |
| OTU849 | 0.062±0.036 | 0.280±0.157 | | 0.164±0.153 | | 0.050±0.034 | g__*Blautia* |
| OTU345 | 0.023±0.010 | 0.156±0.041** | | 0.118±0.110 | | 0.046±0.014 | f__Ruminococcaceae |
| OTU58 | 0.000±0.000 | 0.000±0.000 | | 0.133±0.133 | | 0.045±0.036 | f__Ruminococcaceae |
| OTU793 | 0.000±0.000 | 0.000±0.000 | | 0.053±0.033 | | 0.045±0.037 | g__*Intestinimonas* |
| OTU335 | 0.000±0.000 | 0.000±0.000 | | 0.220±0.218 | | 0.045±0.019 | f__Christensenellaceae |
| OTU622 | 0.002±0.002 | 0.011±0.009 | | 0.104±0.070 | | 0.044±0.032 | s__*Corynebacterium*_*testudinoris* |
| OTU531 | 0.066±0.066 | 0.101±0.079 | | 0.234±0.131 | | 0.040±0.035 | g__*Subdoligranulum* |
| OTU599 | 0.188±0.188 | 0.001±0.001 | | 0.009±0.008 | | 0.039±0.038 | f__S24-7 |
| OTU414 | 0.143±0.105 | 0.069±0.026 | | 0.036±0.022 | | 0.034±0.017 | g__Incertae_Sedis |
| OTU453 | 0.008±0.008 | 0.331±0.293 | | 0.012±0.011 | | 0.033±0.032 | g__*Odoribacter* |
| OTU87 | 0.000±0.000 | 0.000±0.000 | | 0.061±0.048 | | 0.032±0.011 | f__Ruminococcaceae |
| OTU859 | 0.000±0.000 | 0.000±0.000 | | 0.187±0.168 | | 0.032±0.022 | g__*Roseburia* |
| OTU820 | 0.379±0.175 | 1.552±0.783 | | 0.017±0.007 | | 0.031±0.019 | s__*Lactobacillus*_*johnsonii* |
| OTU435 | 0.164±0.063 | 0.030±0.009 | | 0.006±0.004 | | 0.031±0.031 | g__*Hydrogenoanaerobacterium* |
| OTU122 | 0.000±0.000 | 0.015±0.010 | | 0.075±0.045 | | 0.030±0.019 | g__*Peptococcus* |
| OTU596 | 0.000±0.000 | 0.009±0.009 | | 1.334±1.314 | | 0.030±0.018 | f__S24-7 |
| OTU690 | 0.049±0.024 | 0.118±0.047 | | 0.042±0.021 | | 0.029±0.015 | g__Incertae_Sedis |
| OTU42 | 0.025±0.020 | 0.095±0.035 | | 0.200±0.113 | | 0.027±0.007 | f__Ruminococcaceae |
| OTU684 | 0.004±0.004 | 0.358±0.358 | | 0.006±0.003 | | 0.026±0.023 | f__Prevotellaceae |
| OTU208 | 0.000±0.000 | 0.101±0.075 | | 0.040±0.027 | | 0.025±0.017 | f__Ruminococcaceae |
| OTU55 | 1.362±0.896 | 1.356±0.471 | | 0.002±0.002 | | 0.024±0.013 | s__*Clostridium*_*perfringens*_NCTC_8239 |
| OTU301 | 0.217±0.172 | 0.144±0.048 | | 0.009±0.003 | | 0.023±0.011 | g__*Actinobacillus* |
| OTU61 | 3.038±1.624 | 0.220±0.075 | | 0.001±0.001 | | 0.023±0.018 | g__*Butyricimonas* |
| OTU620 | 0.827±0.677 | 0.456±0.201 | | 0.168±0.166 | | 0.023±0.022 | s__*Ruminococcus*_*torques*_ATCC_27756 |
| OTU595 | 0.000±0.000 | 0.122±0.122 | | 0.022±0.013 | | 0.019±0.017 | f__Prevotellaceae |
| OTU685 | 0.024±0.019 | 0.001±0.001 | | 1.095±0.868 | | 0.018±0.007 | g__*Cloacibacillus* |
| OTU747 | 0.002±0.002 | 0.052±0.043 | | 0.039±0.023 | | 0.018±0.011 | f__Ruminococcaceae |
| OTU383 | 0.128±0.075 | 0.188±0.092 | | 0.020±0.009 | | 0.018±0.009 | f__S24-7 |
| OTU686 | 0.257±0.108 | 0.111±0.031 | | 0.104±0.056 | | 0.017±0.012 | f__Ruminococcaceae |
| OTU160 | 0.042±0.023 | 0.537±0.245 | | 0.049±0.033 | | 0.017±0.008 | s__*Oscillibacter*_sp._G2 |
| OTU415 | 0.000±0.000 | 0.000±0.000 | | 0.169±0.169 | | 0.017±0.009 | g__*Alloprevotella* |
| OTU573 | 0.085±0.049 | 0.133±0.022 | | 0.061±0.024 | | 0.015±0.012 | g__*Streptococcus* |
| OTU671 | 0.120±0.118 | 0.008±0.003 | | 0.006±0.005 | | 0.013±0.006 | g__*Oscillibacter* |
| OTU784 | 0.001±0.001 | 0.004±0.003 | | 0.239±0.238 | | 0.013±0.013 | g__*Blautia* |
| OTU130 | 0.000±0.000 | 0.008±0.008 | | 0.095±0.052 | | 0.011±0.009 | f__Family_XIII |
| OTU809 | 0.113±0.105 | 0.098±0.068 | | 0.008±0.004 | | 0.010±0.006 | g__*Alistipes* |
| OTU190 | 0.058±0.058 | 0.000±0.000 | | 0.006±0.006 | | 0.008±0.007 | g__RC9_gut_group |
| OTU499 | 0.000±0.000 | 0.000±0.000 | | 2.210±2.208 | | 0.008±0.007 | g__*Anaerovibrio* |
| OTU226 | 0.010±0.009 | 0.005±0.005 | | 0.124±0.074 | | 0.008±0.006 | s__*Porphyromonadaceae*_*bacterium*_DJF_B175 |
| OTU424 | 0.114±0.080 | 0.096±0.033 | | 0.011±0.003 | | 0.008±0.004 | g__*Streptococcus* |
| OTU631 | 0.000±0.000 | 0.000±0.000 | | 0.073±0.048 | | 0.008±0.003 | f__Family_XIII |
| OTU418 | 0.056±0.037 | 0.119±0.040 | | 0.000±0.000 | | 0.007±0.006 | g__*Butyricimonas* |
| OTU115 | 0.000±0.000 | 0.003±0.003 | | 0.093±0.074 | | 0.007±0.002 | f__Prevotellaceae |
| OTU445 | 0.469±0.256 | 0.202±0.097 | | 0.001±0.001 | | 0.007±0.006 | g__*Bacteroides* |
| OTU566 | 0.000±0.000 | 0.000±0.000 | | 0.090±0.078 | | 0.007±0.007 | s__*Brachyspira*_sp._NSH-25 |
| OTU8 | 0.000±0.000 | 0.008±0.006 | | 0.081±0.081 | | 0.006±0.003 | f__Ruminococcaceae |
| OTU196 | 0.953±0.431 | 0.382±0.232 | | 0.000±0.000 | | 0.006±0.004 | g__*Bacteroides* |
| OTU310 | 0.046±0.018 | 0.132±0.053 | | 0.004±0.004 | | 0.006±0.003 | s__*Actinobacillus*_*minor* |
| OTU264 | 0.095±0.074 | 0.200±0.114 | | 0.015±0.012 | | 0.006±0.003 | s__*Coprococcus*_sp._HPP0074 |
| OTU783 | 0.037±0.037 | 0.001±0.001 | | 0.070±0.056 | | 0.005±0.004 | g__*Eubacterium* |
| OTU192 | 0.327±0.207 | 1.720±1.438 | | 0.002±0.001 | | 0.005±0.004 | s__*Pasteurella*_*aerogenes* |
| OTU211 | 0.271±0.073 | 0.573±0.284 | | 0.058±0.051 | | 0.005±0.004 | g__*Veillonella* |
| OTU483 | 5.586±1.912 | 2.732±1.605 | | 0.002±0.002 | | 0.005±0.002 | s__*Bacteroides*_*thetaiotaomicron* |
| OTU855 | 0.000±0.000 | 0.005±0.005 | | 0.158±0.158 | | 0.004±0.003 | f__Ruminococcaceae |
| OTU396 | 0.144±0.144 | 0.000±0.000 | | 0.000±0.000 | | 0.004±0.003 | o__Gastranaerophilales |
| OTU551 | 0.007±0.004 | 0.135±0.096 | | 0.057±0.046 | | 0.004±0.004 | s__*Lactobacillus*_*mucosae* |
| OTU303 | 0.040±0.033 | 0.176±0.098 | | 0.002±0.001 | | 0.004±0.004 | g__*Lactobacillus* |
| OTU773 | 0.141±0.139 | 0.005±0.003 | | 0.037±0.030 | | 0.004±0.004 | f__Lachnospiraceae |
| OTU77 | 0.000±0.000 | 0.055±0.055 | | 0.000±0.000 | | 0.004±0.004 | f__Family_XIII |
| OTU420 | 0.056±0.040 | 0.019±0.007 | | 0.003±0.003 | | 0.003±0.003 | g__*Anaerotruncus* |
| OTU439 | 0.010±0.003 | 0.055±0.029 | | 0.008±0.003 | | 0.003±0.002 | g__*Actinomyces* |
| OTU564 | 0.636±0.636 | 0.000±0.000 | | 0.003±0.003 | | 0.003±0.002 | g__*Spirochaeta* |
| OTU625 | 0.024±0.008 | 0.069±0.030 | | 0.029±0.010 | | 0.003±0.002* | g__*Peptostreptococcus* |
| OTU722 | 0.086±0.067 | 0.175±0.104 | | 0.002±0.001 | | 0.002±0.002 | g__*Haemophilus* |
| OTU154 | 0.016±0.016 | 1.763±0.104* | | 3.289±3.254 | | 0.002±0.002 | f__Prevotellaceae |
| OTU385 | 0.000±0.000 | 0.106±0.081 | | 0.003±0.002 | | 0.002±0.002 | s__*Odoribacter*_*splanchnicus*_DSM_220712 |
| OTU492 | 0.175±0.161 | 0.021±0.009 | | 0.002±0.002 | | 0.002±0.002 | s__*Porphyromonadaceae*_*bacterium*_AIP925.11 |
| OTU562 | 0.000±0.000 | 0.000±0.000 | | 0.097±0.097 | | 0.002±0.002 | f__Ruminococcaceae |
| OTU571 | 0.251±0.251 | 0.002±0.002 | | 0.006±0.005 | | 0.002±0.002 | s__*Parabacteroides*_*chinchillae* |
| OTU426 | 1.143±1.073 | 0.042±0.020 | | 0.009±0.006 | | 0.002±0.001 | g__*Pseudoflavonifractor* |
| OTU621 | 2.052±1.247 | 0.103±0.047 | | 0.021±0.013 | | 0.002±0.001 | g__*Alistipes* |
| OTU682 | 0.001±0.001 | 1.970±1.968 | | 0.538±0.538 | | 0.002±0.001 | g__*Alloprevotella* |
| OTU120 | 0.050±0.048 | 0.096±0.048 | | 0.005±0.005 | | 0.001±0.001 | g__*Lactobacillus* |
| OTU442 | 0.000±0.000 | 0.367±0.267 | | 0.001±0.001 | | 0.001±0.001 | s__*Bacteroide*s_*stercoris*_ATCC_43183 |
| OTU772 | 0.044±0.019 | 0.053±0.023 | | 0.001±0.001 | | 0.001±0.001 | g__*Anaerotruncus* |
| OTU82 | 0.000±0.000 | 0.007±0.007 | | 0.071±0.044 | | 0.001±0.001 | g__*Bacteroides* |
| OTU113 | 1.379±1.366 | 1.322±1.125 | | 0.001±0.001 | | 0.001±0.001 | g__*Alloprevotella* |
| OTU594 | 0.364±0.312 | 0.037±0.018 | | 0.034±0.024 | | 0.001±0.001 | s__*Intestinimonas*_*butyriciproducens* |
| OTU759 | 0.915±0.773 | 0.194±0.083 | | 0.000±0.000 | | 0.001±0.001 | f__Ruminococcaceae |
| OTU23 | 0.144±0.144 | 0.000±0.000 | | 0.000±0.000 | | 0.000±0.000 | g__*Fusobacterium* |
| OTU38 | 0.000±0.000 | 0.000±0.000 | | 1.439±1.439 | | 0.000±0.000 | f__Ruminococcaceae |
| OTU75 | 0.333±0.181 | 0.031±0.021 | | 0.000±0.000 | | 0.000±0.000 | g__*Bacteroides* |
| OTU89 | 0.006±0.003 | 0.066±0.045 | | 0.001±0.001 | | 0.000±0.000 | s__*Bacteroides*_*nordii* |
| OTU104 | 0.173±0.172 | 0.018±0.011 | | 0.000±0.000 | | 0.000±0.000 | f__vadinBB60 |
| OTU142 | 0.296±0.173 | 0.073±0.029 | | 0.000±0.000 | | 0.000±0.000 | g__*Butyricimonas* |
| OTU231 | 0.107±0.063 | 0.043±0.027 | | 0.001±0.001 | | 0.000±0.000 | f__Clostridiaceae_1 |
| OTU236 | 0.000±0.000 | 0.001±0.001 | | 0.002±0.001 | | 0.000±0.000 | s__*Clostridiales*_*bacterium*_canine_oral_taxon_100 |
| OTU263 | 0.001±0.001 | 0.744±0.723 | | 0.000±0.000 | | 0.000±0.000 | g__*Bacteroides* |
| OTU280 | 5.177±5.172 | 0.269±0.266 | | 0.000±0.000 | | 0.000±0.000 | g__*Prevotella* |
| OTU290 | 0.423±0.423 | 0.391±0.367 | | 0.000±0.000 | | 0.000±0.000 | s__*Bacteroides*_*pyogenes* |
| OTU344 | 0.351±0.254 | 0.852±0.800 | | 0.000±0.000 | | 0.000±0.000 | g__*Akkermansia* |
| OTU347 | 0.149±0.149 | 0.740±0.738 | | 0.038±0.038 | | 0.000±0.000 | g__*Phascolarctobacterium* |
| OTU361 | 0.289±0.289 | 0.000±0.000 | | 0.000±0.000 | | 0.000±0.000 | g__*Spirochaeta* |
| OTU382 | 0.076±0.076 | 0.000±0.000 | | 0.000±0.000 | | 0.000±0.000 | g__*Bacteroides* |
| OTU389 | 0.004±0.004 | 0.000±0.000 | | 0.124±0.124 | | 0.000±0.000 | f__Ruminococcaceae |
| OTU411 | 0.000±0.000 | 1.499±1.410 | | 0.000±0.000 | | 0.000±0.000 | g__*Succiniclasticum* |
| OTU459 | 0.000±0.000 | 0.000±0.000 | | 0.289±0.288 | | 0.000±0.000 | g__*Synergistes* |
| OTU538 | 0.000±0.000 | 0.000±0.000 | | 0.082±0.082 | | 0.000±0.000 | o__RF9 |
| OTU585 | 0.000±0.000 | 0.075±0.075 | | 0.000±0.000 | | 0.000±0.000 | g__*Intestinimonas* |
| OTU619 | 0.000±0.000 | 0.008±0.008 | | 0.074±0.073 | | 0.000±0.000 | g__*Anaerovibrio* |
| OTU710 | 0.000±0.000 | 0.512±0.512 | | 0.000±0.000 | | 0.000±0.000 | g__*Succiniclasticum* |
| OTU711 | 0.000±0.000 | 0.343±0.340 | | 0.007±0.004 | | 0.000±0.000 | g__*Anaerovibrio* |
| OTU721 | 3.958±3.868 | 0.017±0.013 | | 0.009±0.008 | | 0.000±0.000 | s__*Bacteroides*_*fluxus* |
| OTU737 | 0.596±0.270 | 0.712±0.388 | | 0.000±0.000 | | 0.000±0.000 | g__*Bacteroides* |
| OTU752 | 0.000±0.000 | 0.000±0.000 | | 0.078±0.075 | | 0.000±0.000 | o__Gastranaerophilales |
| OTU761 | 0.363±0.346 | 0.288±0.180 | | 0.009±0.009 | | 0.000±0.000 | g__*Phascolarctobacterium* |
| OTU776 | 0.078±0.040 | 0.011±0.006 | | 0.000±0.000 | | 0.000±0.000 | s__*Flavonifractor*_*plautii* |
| OTU818 | 0.027±0.019 | 0.169±0.119 | | 0.002±0.001 | | 0.000±0.000 | g__*Lactobacillus* |

## ^1^OTUs with relative abundances higher than 0.05% within total bacteria were sorted and showed in the table.

^2^The consensus sequence of each OTU was annotated to the closest lineage using MOTHUR program against the SILVA 16S rRNA reference database. s = species; g = genus; f = family; o = order**.**

## * means the significantly difference (*P* < 0.05) between SB group and CO group.** means the significantly difference (*P* < 0.01) between SB group and CO group.
